# Jujube witches’ broom phytoplasmas inhibit ZjBRC1-mediated abscisic acid metabolism to induce shoot proliferation

**DOI:** 10.1093/hr/uhad148

**Published:** 2023-07-24

**Authors:** Fuli Ma, Shanqi Zhang, Yu Yao, Mengting Chen, Ning Zhang, Mingsheng Deng, Wei Chen, Chi Ma, Xinyue Zhang, Chenglong Guo, Xiang Huang, Zhenyuan Zhang, Yamei Li, Tingyi Li, Junyong Zhou, Qibao Sun, Jun Sun

**Affiliations:** College of Horticulture, Anhui Agricultural University, 130 West Changjiang Road, Hefei City 230036, Anhui Province, China; College of Horticulture, Anhui Agricultural University, 130 West Changjiang Road, Hefei City 230036, Anhui Province, China; College of Horticulture, Anhui Agricultural University, 130 West Changjiang Road, Hefei City 230036, Anhui Province, China; State Key Laboratory of Tea Plant Biology and Utilization, Anhui Agricultural University, 130 West Changjiang Road, Hefei City 230036, Anhui Province, China; College of Horticulture, Anhui Agricultural University, 130 West Changjiang Road, Hefei City 230036, Anhui Province, China; State Key Laboratory of Tea Plant Biology and Utilization, Anhui Agricultural University, 130 West Changjiang Road, Hefei City 230036, Anhui Province, China; College of Horticulture, Anhui Agricultural University, 130 West Changjiang Road, Hefei City 230036, Anhui Province, China; College of Horticulture, Anhui Agricultural University, 130 West Changjiang Road, Hefei City 230036, Anhui Province, China; College of Horticulture, Anhui Agricultural University, 130 West Changjiang Road, Hefei City 230036, Anhui Province, China; College of Horticulture, Anhui Agricultural University, 130 West Changjiang Road, Hefei City 230036, Anhui Province, China; College of Horticulture, Anhui Agricultural University, 130 West Changjiang Road, Hefei City 230036, Anhui Province, China; College of Horticulture, Anhui Agricultural University, 130 West Changjiang Road, Hefei City 230036, Anhui Province, China; College of Horticulture, Anhui Agricultural University, 130 West Changjiang Road, Hefei City 230036, Anhui Province, China; College of Horticulture, Anhui Agricultural University, 130 West Changjiang Road, Hefei City 230036, Anhui Province, China; College of Horticulture, Anhui Agricultural University, 130 West Changjiang Road, Hefei City 230036, Anhui Province, China; State Key Laboratory of Tea Plant Biology and Utilization, Anhui Agricultural University, 130 West Changjiang Road, Hefei City 230036, Anhui Province, China; College of Horticulture, Anhui Agricultural University, 130 West Changjiang Road, Hefei City 230036, Anhui Province, China; College of Horticulture, Anhui Agricultural University, 130 West Changjiang Road, Hefei City 230036, Anhui Province, China; Horticulture Research Institute, Anhui Academy of Agricultural Sciences, 40 South Nongke Road, Hefei City 230031, Anhui Province, China; Horticulture Research Institute, Anhui Academy of Agricultural Sciences, 40 South Nongke Road, Hefei City 230031, Anhui Province, China; College of Horticulture, Anhui Agricultural University, 130 West Changjiang Road, Hefei City 230036, Anhui Province, China

## Abstract

Jujube witches’ broom (JWB) phytoplasmas parasitize the sieve tubes of diseased phloem and cause an excessive proliferation of axillary shoots from dormant lateral buds to favour their transmission. In previous research, two JWB effectors, SJP1 and SJP2, were identified to induce lateral bud outgrowth by disrupting ZjBRC1-mediated auxin flux. However, the pathogenesis of JWB disease remains largely unknown. Here, tissue-specific transcriptional reprogramming was examined to gain insight into the genetic mechanisms acting inside jujube lateral buds under JWB phytoplasma infection. JWB phytoplasmas modulated a series of plant signalling networks involved in lateral bud development and defence, including auxin, abscisic acid (ABA), ethylene, jasmonic acid, and salicylic acid. JWB-induced bud outgrowth was accompanied by downregulation of ABA synthesis within lateral buds. ABA application rescued the bushy appearances of transgenic *Arabidopsis* overexpressing *SJP1* and *SJP2* in Col-0 and *ZjBRC1* in the *brc1-2* mutant. Furthermore, the expression of *ZjBRC1* and ABA-related genes *ZjHB40* and *ZjNCED3* was negatively correlated with lateral main bud outgrowth in decapitated healthy jujube. Molecular evidence showed that ZjBRC1 interacted with ZjBRC2 via its N-terminus to activate *ZjHB40* and *ZjNCED3* expression and ABA accumulation in transgenic jujube calli. In addition, *ZjBRC1* widely regulated differentially expressed genes related to ABA homeostasis and ABA signalling, especially by binding to and suppressing ABA receptors. Therefore, these results suggest that JWB phytoplasmas hijack the *ZjBRC1*-mediated ABA pathways to stimulate lateral bud outgrowth and expansion, providing a strategy to engineer plants resistant to JWB phytoplasma disease and regulate woody plant architecture to promote crop yield and quality.

## Introduction

Chinese jujube (*Ziziphus jujuba* Mill.), one of the most economically and ecologically important species in the Rhamnaceae family, was first domesticated in the Shanxi–Shaanxi area [1] and has subsequently been grown in arid and semiarid areas of China for >7000 years [2]. As a traditional herbal medicine and additive in functional food products [3, 4], jujube has been introduced worldwide to promote the development of the modern jujube industry [2]. Although China has abundant germplasm resources with ~930 jujube cultivars [2], most of them are sensitive to jujube witches’ broom (JWB) disease [5], which is a ‘jujube cancer’ and the most devastating disease caused by JWB phytoplasma infection. Since JWB phytoplasmas are difficult to culture *in vitro*, little information about the interaction between jujube and JWB phytoplasmas is available.

The first report of JWB disease in China dates back to 1942, and virus-like particles were observed in diseased leaves 32 years later [6]. The causal agent was confirmed to be a member of the 16SrV-B group from the genus ‘*Candidatus* Phytoplasma’ [7] and was highly conserved with strains identified in Korea and Japan [8]. JWB phytoplasma contains a small genome that encodes 694 proteins [9]; however, it lacks many metabolic pathways essential for survival *in vitro*. Thus, this bacterium parasitizes the sieve tubes of diseased phloem [5] and is annually and dynamically redistributed among different tissues, especially in the young bearing shoot and leaf stalk [10]. Phytoplasmas manipulate host vegetative and reproductive growth to allow insect colonization, which is their most important transmission method [11–14]. However, the obligate parasites in jujube are deleterious to host plant development. JWB phytoplasma-infected jujube plants show excessive growth of dormant lateral main buds, yellowing, and a bushy appearance with damaged chloroplast and sieve-element structures, disrupted sugar metabolism, and altered photosynthetic responses [15, 16], which subsequently leads to their death within several years.

Comprehensively controlling JWB disease is extremely challenging for jujube breeders. Removal of the diseased branches, root offshoots and even whole plants is frequently performed but is not the best therapeutic approach as it is laborious and time-consuming. Antibiotics by trunk injection (such as oxytetracycline hydrochloride and tetracycline hydrochloride) were used to cure diseased trees before the phytoplasma concentration reached its peak [5]. Since jujube plants could be asymptomatic at the early stage of JWB phytoplasma infection and the distribution of JWB phytoplasmas among different seasons and tissues was uneven, researchers were unable to determine the optimal injection period. Screening of JWB-resistant germplasms [5, 17] and cryopreservation-mediated plantlet regeneration [18] would be effective for JWB disease management and long-term storage of phytoplasma-free jujube plants. Furthermore, in the past several years, information on jujube–JWB phytoplasma interactions has been obtained by multiomics analyses, highlighting some special gene loci and hormone pathways related to defence responses in diseased leaves [19–21]. Fully understanding the pathogenesis of phytoplasma disease would facilitate a precise breeding strategy towards engineering plants resistant to JWB phytoplasma infection.

Phytoplasmas directly secrete effectors that target and destabilize host growth regulators to induce witches’ broom symptoms [11–14]. Secreted AY-WB protein 11 (SAP11) from aster yellows phytoplasma strain witches’ broom (AY-WB) induced morphological changes in leaf shape and stem proliferation [22] by destabilization of CIN-TCP transcription factors [23]. Subsequently, SAP11 homologues were found to induce witches’ broom disease in different phytoplasma-infected plants, including apple [24], wheat [25], peanut [26], maize [27], and lime [14]. SAP05, another member of the SAP family, modulated host lifespan and induced witches’ broom-like proliferation by mediating the degradation of SQUAMOSA-PROMOTER BINDING PROTEIN-LIKE (SPL) and W-GATA-R-binding (GATA, W = T or A; R = G or A) developmental regulators [13]. In addition, the PaWB-SAP54 effector from *Paulownia* witches’ broom (PaWB) phytoplasma, which is a homologue of the leaf-like flower-induced protein SAP54 from AY-WB [12, 28], induced the formation of secondary branches by mediating the degradation of SPLa in a ubiquitin-dependent pathway in *Populus trichocarpa* [29]. These findings indicated that the pathogenesis of witches’ broom caused by phytoplasma infection extends far beyond current knowledge in woody plants.

In our previous research, the lateral main buds from 1-year-old primary extension shoots transitioned from dormancy to outgrowth during the early stage of JWB phytoplasma infection [30]. Two secreted JWB proteins, 1 and 2 (SJP1 and SJP2), were confirmed to induce an increase in lateral branches by directly targeting and mediating the destabilization of ZjBRC1, which negatively regulated auxin efflux in the lateral main buds [30]. An interesting observation was that the infected dormant buds were activated or in an ‘idling’ mode that should not be considered dormant [30]. JWB phytoplasmas might disturb multi-factors to collectively induce buds to prepare for outgrowth and expansion. To better understand the genetic mechanisms acting inside the lateral main buds under JWB phytoplasma infection, tissue-specific transcriptional changes were investigated. JWB phytoplasmas modulate a series of plant signalling networks, including auxin (IAA), abscisic acid (ABA), ethylene, jasmonic acid (JA), and salicylic acid (SA). In addition, we provided evidence that JWB phytoplasmas hijacked the *ZjBRC1*-mediated ABA pathway to stimulate lateral bud outgrowth.

## Results

### Transcriptional dynamics of lateral main bud development in jujube witches’ broom phytoplasma-infected jujube plants

**Figure 1:**
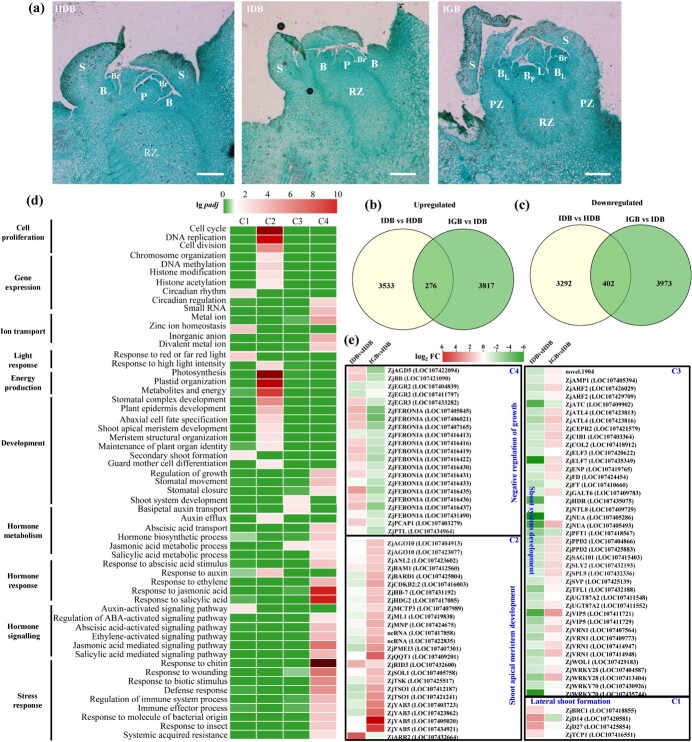
Transcriptional profiling of lateral main bud development infected by JWB phytoplasmas. (a) Longitudinal sections of the developing lateral main bud in 1-year-old primary extension shoots of healthy and JWB-infected jujube plants. HDB, healthy dormant bud; IDB, infected dormant bud; IGB, infected growing bud; B, secondary bud primordium; B_L_, secondary bud; B_P_, primary bud; Br, bract; L, leaf primordium; P, primary bud primordium; PZ, peripheral zone; RZ, rib zone; S, bud scale. Scale bars = 100 μm. (b, c) Venn diagrams showing the upregulated (b) and downregulated (c) DEGs between IDB and HDB and between IGB and IDB. (d) Over-represented GO terms of DEGs according to the co-expression clusters. GO terms with adjusted *P*-value (*P*_adj_) ≤ 0.05 (−log*P*_adj_ ≥ 1.3) were considered significantly enriched. (e) Expression profiles of DEGs involved in negative regulation of growth, shoot apical meristem development, shoot system development, and secondary shoot formation.

The dormancy of lateral main buds was released to give rise to lateral branches once JWB phytoplasma infection occurred [30]. To determine the difference in bud development, the ultrastructures of the lateral main buds were compared between 1-year-old primary extension shoots of healthy and JWB-infected jujube plants. The axillary meristem of the healthy dormant bud (HDB) and the infected dormant bud (IDB) included a primary bud primordium (P), two secondary bud primordia (B) and an underlying rib zone (RZ), which were enclosed within two thick bud scales (Fig. 1a). In the infected growing bud (IGB), the rib zone together with the peripheral zone displaced downwards to form the stem, and the primary bud primordium and secondary bud primordia developed into primary and secondary buds, respectively (Fig. 1a).

To comprehensively clarify the regulatory mechanism of lateral main bud outgrowth induced by JWB phytoplasma infection, tissue-specific RNA-seq was performed to investigate the transcriptional dynamics. Compared with IDB, a total of 12 376 unique genes were differentially expressed (Fig. S1a, Supplementary Data Table S2), of which 7503 and 8468 were differentially regulated in HDB and IGB, respectively (Supplementary Data Fig. S1b). The majority (91–92%) of the up- and downregulated genes were uniquely expressed in HDB and IGB (Fig. 1b and c). Four gene coexpression clusters were identified according to the expression profiles of the unique genes (Supplementary Data Fig. S1c). Genes in Clusters 1 and 2 showed opposite patterns in HDB and IDB but had consistently high expression in IGB; these clusters likely contain genes that promote rapid growth and expansion when dormancy is bypassed. Genes in Cluster 3 showed opposite patterns in IGB and HDB but had consistently low expression in IDB; this cluster likely includes genes that are associated with shoot system development. In contrast, the genes in Cluster 4 showed consistently low expression in IGB but opposite patterns in HDB and IDB; this cluster most likely contains genes that dynamically participated in the regulation of bud dormancy or outgrowth (Supplementary Data Fig. S1c).

Gene Ontology (GO) enrichment analysis showed that Clusters 1–4 were specifically annotated into several distinct physiological processes (Fig. 1d and Supplementary Data Table S3). Cluster 1 was enriched for genes involved in zinc ion homeostasis, response to red or far red light, circadian rhythm, auxin-activated signalling pathway, and secondary shoot formation. Cluster 2 was enriched for genes involved in responses to cell proliferation, gene expression, energy production, and development. Genes that participated in shoot system development and JA metabolic processes were specifically grouped in Cluster 3. Furthermore, Cluster 4 was enriched for genes involved in responses to hormone metabolism and signalling, stress response, and development. In particular, genes involved in the metabolism and signalling of ethylene, IAA, ABA, JA, and SA were identified in Cluster 4, indicating that these hormones played important roles in the regulation of jujube–phytoplasma interactions and lateral main bud development.

Interestingly, a dynamic reprogramming of differentially expressed genes (DEGs) towards bud outgrowth and lateral shoot formation was found for Clusters 1–4 (Fig. 1e). *ZjD27* (*DWARF 27*), *ZjD14* (*DWARF 14*), *ZjBRC1*, and *ZjTCP1* in Cluster 1, four homologs involved in the strigolactone pathway [31], were upregulated in IDB and IGB. A similar pattern was observed for the homologs of *ARGONAUTE10* (*ZjAGO10*, LOC107404913 and LOC107423077) in Cluster 2, which promoted axillary meristem development [32]. *ZjSVP* (LOC107425139) in Cluster 3, a homolog of *SHORT VEGETATIVE PHASE-LIKE* (*SVL*), which was known to be a negative regulator of bud break in hybrid aspen [33], was downregulated in IDB and IGB as well as a homolog of trihelix transcription factor *PETAL LOSS* (*ZjPTL*, LOC107434964) in Cluster 4, which was involved in limiting lateral growth of organs. Taken together, the results suggest that JWB phytoplasmas stimulated lateral buds to transition from dormancy to outgrowth, possibly through the comprehensive disruption of hormones, energy supply, and cell proliferation exactly as these were both necessary and sufficient for normal bud outgrowth.

### ABA synthesis is downregulated in jujube witches’ broom phytoplasma-infected lateral main buds

Coincidentally, DEGs involved in the auxin and ABA (Supplementary Data Table S4) but not gibberellin signalling pathways were enriched in Cluster 1 and Cluster 4 (Fig. 1d), providing new evidence that JWB phytoplasmas manipulate these hormone signalling pathways to regulate lateral main bud outgrowth. Considering that ZjBRC1 controls auxin efflux channels in jujube lateral main buds [30], 13 DEGs were identified and involved in auxin transport. *ZjPIN1c* and *ZjPIN3* were directly targeted and inhibited by ZjBRC1 [30]. *ZjWAT1*, which encoded a vacuolar auxin transport facilitator, was required for auxin homoeostasis [34]. All these genes were upregulated in IGB, where the ZjBRC1 protein was absent [30]. Thus, *ZjBRC1* not only modulated auxin efflux but also participated in auxin homoeostasis to promote auxin accumulation in dormant buds.

**Figure 2:**
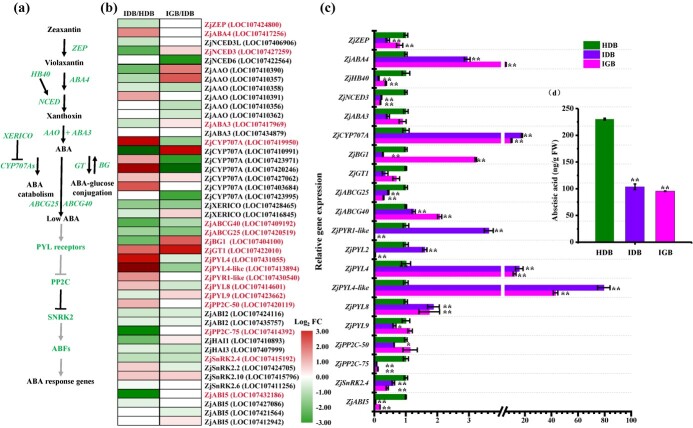
JWB phytoplasmas suppressed ABA synthesis in developing lateral main buds. (a) Model of ABA homeostasis and core signalling pathway. (b) Heat map of DEGs involved in the ABA biosynthesis, catabolism, transport, and signalling pathways in healthy and JWB phytoplasma-infected lateral main buds. The colour gradient scale indicates the log value of expression fold change (log_2_FC). HDB, healthy dormant bud; IDB, infected dormant bud; IGB, infected growing bud. (c) qRT–PCR analysis confirmed expressions of the selected ABA-related DEGs. Error bars show standard deviations from three independent experiments (^**^P < .01, ^*^P < .05; Student’s *t*-test). (d) Endogenous ABA content in healthy and JWB phytoplasma-infected lateral main buds. Approximately 100 mg of HDB, IDB, and IGB from JWB-infected and healthy primary extension shoots was collected, respectively. Error bars show standard deviations from three independent experiments (Tukey’s *post hoc* test, *P* < .05).

In addition, ABA synthesis genes, including three *NINE-CIS-EPOXYCAROTENOID DIOXYGENASE*s (*ZjNCED*s, Supplementary Data Fig. S2a and b), five abscisic aldehyde oxidases (*ZjAAO*s) and two *ABA DEFICIENT3*s (*ZjABA3*s), were downregulated in IDB compared with HDB (Fig. 2a and b). The same expression patterns were also observed in ABA degradation repressors (*ZjXERICO*) and ABA transporters (*ZjABCG25/40*), but opposite trends were observed in ABA catabolic genes (*ZjCYP707A*s) and ABA-glucose conjugation genes (*ZjGT1*) (Fig. 2a and b). Furthermore, *ZjHB40*, a *HOMEOBOX PROTEIN 40* (*AtHB40*) homologue that directly targets *AtNCED3* to promote ABA accumulation in *Arabidopsis* axillary buds [35] was downregulated in IDB and IGB (Fig. 2c). We next assessed ABA production in healthy and JWB phytoplasma-infected lateral main buds (Fig. 2d). ABA accumulation was significantly reduced in IDB and IGB, which was correlated with the rapid growth and expansion of lateral buds in JWB phytoplasma-infected jujube plants (Fig. 2d). Taken together, the results suggest that JWB phytoplasma infection might inhibit ABA biosynthesis, thereby inducing bud outgrowth from dormancy.

### Jujube witches’ broom phytoplasmas inhibited ZjBRC1 to control lateral shoot branching

**Figure 3:**
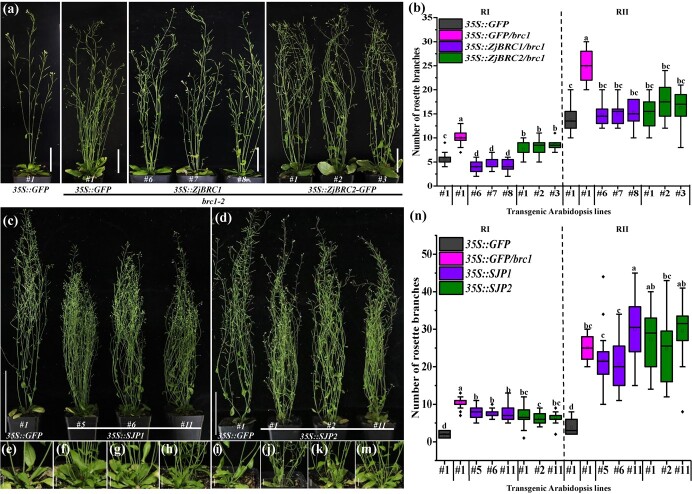
Shoot branching phenotype of *ZjBRC1*- and *ZjBRC2*-transgenic *Arabidopsis* lines. (a) Representative images of *T*_2_*35S::GFP* transgenic *Arabidopsis* and *brc1-2* mutants carrying *35S::GFP*, *35S::ZjBRC1-GFP* and *35S::ZjBRC2-GFP*. Transgenic lines were grown in MS medium for 12 days and subsequently grown for 30 days under long-day conditions. (b) Quantitative analysis of rosette-leaf branches of *35S::ZjBRC1/brc1-2* and *35S::ZjBRC2/brc1-2* transgenic lines at 30 days after transplanting from MS medium. Error bars represent standard deviations, *n* = 12. Representative images (c) and close-up views (e–h) of rosette leaf branches of *T*_2_*35S::SJP1* transgenic *Arabidopsis* lines. Representative images (d) and close-up views (i–m) of rosette leaf branches of *T*_2_*35S::SJP2* transgenic *Arabidopsis* lines. Transgenic lines were grown in MS medium for 12 days and subsequently grown for 42 days under long-day conditions. (n) Quantitative analysis of rosette-leaf branches of *35S::SJP1* and *35S::SJP2* transgenic lines at 42 days after transplanting from MS medium. Error bars represent standard deviations (*n* = 20). Different letters indicate significant differences among means as determined using one-way ANOVA followed by Tukey’s *post hoc* test (*P* < .05).

BRC1 is well-known as a signal integrator that represses bud outgrowth by controlling *BRC1*-dependent gene-regulatory networks [36], such as positively regulating ABA biosynthesis in *Arabidopsis* [31, 35, 37]. In our previous research, the accumulation of ZjBRC1 was decreased in IGB as well as in *35S::SJP1*- and *35S::SJP2*-transgenic jujube calli, which were obtained via *Agrobacterium*-mediated transformation [30]. These SJP effectors might hijack ZjBRC1 to control ABA biosynthesis to induce lateral shoot branching. To this end, *ZjBRC1* and *ZjBRC2* were firstly overexpressed in *brc1-2 Arabidopsis* mutants (Fig. 3a). Six weeks after sowing, *35S::GFP/brc1-2* lines showed increased bud outgrowth and produced significantly more primary (RI) and secondary (RII) rosette-leaf branches than *35S::GFP* lines (Fig. 3b). In contrast, the *35S::ZjBRC1/brc1-2* and *35S::ZjBRC2/brc1-2* lines had significantly lower numbers of RIs and RIIs than the *35S::GFP/brc1-2* lines, and the total number of rosette branches was similar to that of the *35S::GFP* lines (Fig. 3b). Furthermore, the numbers of primary cauline-leaf branches (CI) showed no difference among all the lines, and decreased numbers of secondary cauline-leaf branches (CII) were observed in the *35S::GFP/brc1-2*, *35S::ZjBRC1/brc1-2* and *35S::ZjBRC2/brc1-2* lines compared with the *35S::GFP* lines (Supplementary Data Fig. S3). These results indicated that *ZjBRC1* played roles in the suppression of lateral shoot branching in *Arabidopsis*.

To determine whether JWB phytoplasma effectors induced the increase in lateral branches, *SJP1* and *SJP2* were further stably expressed in *Arabidopsis* (Fig. 3c–m). The bushy appearances of the *35S::SJP1* and *35S::SJP2* lines were similar to those of the *35S::GFP/brc1-2* lines but opposite to the appearances of the *35S::ZjBRC1/brc1-2* and *35S::ZjBRC2-/brc1-2* lines (Fig. 3c–m), which showed a significant increase in the numbers of RIs and RIIs (Fig. 3n). The yeast two-hybrid (Y2H) assay showed that both SJP1 and SJP2 interacted with *Arabidopsis* AtBRC1 (Supplementary Data Fig. S4a). AtBRC1 accumulation was significantly suppressed in the presence of SJP1 and SJP2 (Supplementary Data Fig. S4b). These observations indicated that SJP1 and SJP2 could also destabilize AtBRC1 to induce lateral shoot branching in *Arabidopsis*.

### ABA represses the bushy appearances of *SJP1/2* and *brc1-2* transgenic lines

**Figure 4:**
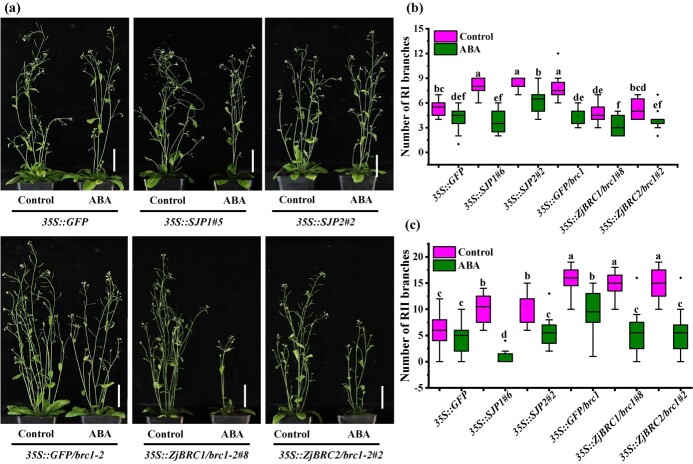
ABA suppresses the excess-branching phenotype of *SJP1/2* and *brc1* mutants. (a) Branching phenotypes of transgenic lines carrying *35S::SJP1*, *35S::SJP2, 35S::ZjBRC1/brc1-2* and *35S::ZjBRC2/brc1-2* treated with or without 50 μM ABA. For branching responses to ABA, the axils of rosette leaves of *T*_2_ transgenic lines were treated with or without 50 μM ABA 13 days after transplanting from MS medium. The treatment was carried out once a day and representative images were photographed 15 days after the treatment. *35S::GFP* and *35S::GFP/brc1-2* lines were used as the control. (b, c) Quantitative analysis of primary (b) and secondary (c) rosette-leaf branches of transgenic lines treated with or without 50 μM ABA 28 days after transplanting from MS medium. Error bars represent standard deviations from three biological replicates (*n* = 12). Different letters indicate significant differences among means as determined using one-way ANOVA followed by Tukey’s *post hoc* test (*P* < .05).

ABA accumulation was significantly reduced in IDB and IGB, which was correlated with the rapid growth and expansion of lateral buds in JWB phytoplasma-infected jujube plants (Fig. 2a). To determine whether the bushy appearances of the *SJP1/2* and *brc1* transgenic lines (Supplementary Data Fig. S5) were positively correlated with the failure to accumulate ABA in buds, 50 μM ABA was directly applied to the axils of rosette leaves every day after bolting, and branch numbers were measured at 15 days after the start of the treatment (Fig. 4a and Supplementary Data Fig. S6). ABA application significantly decreased the numbers of RIs in the *35S::SJP1*, *35S::SJP2* and *brc1-2* background lines but not those in the *35S::GFP* lines (Fig. 4b). In addition, the *35S::SJP1*, *35S::SJP2*, *35S::ZjBRC1/brc1-2*, and *35S::ZjBRC2/brc1-2* lines showed fewer RIIs after the ABA treatment (Fig. 4c). The *35S::GFP* lines showed no significant difference in RIIs after the ABA treatment (Fig. 4c). These results confirmed the possibility that the bushy appearance of *SJP1/2* was partially caused by repressing *ZjBRC1*-controlled ABA accumulation in the buds. It was noticed that there was a large discrepancy in leaf area between plants, which might account for differences in branch numbers [38].

### Expression of *ZjBRC1* and ABA-related genes is negatively correlated with lateral main bud outgrowth in jujube

**Figure 5:**
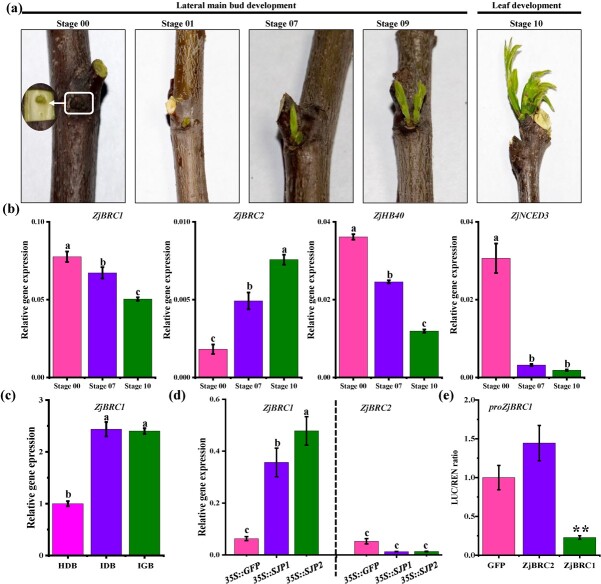
Expression patterns of *ZjBRC1* during lateral bud development and in *SJP1/2* jujube calli. (a) Phenological growth stages of lateral main bud development. The top main buds and secondary shoots of the extension shoots were removed by decapitation to promote bud outgrowth. Stages were determined according to the BBCH scale. (b) Expression of *ZjBRC1* and ABA-marker genes during lateral main bud development. The expression level of each gene was calculated using the 2^−ΔCT^ method. Standard deviations were obtained from three independent biological replicates. (c) Expression of *ZjBRC1* in healthy and JWB phytoplasma-infected lateral main buds. (d) Expression of *ZjBRC1* in *SJP1/2* jujube calli. (e) Dual-luciferase assays showing auto-inhibition of *ZjBRC1*. GFP was used as a negative control. Effector and reporter constructs were co-transformed into *N. benthamiana* leaves, and the ratio of LUC to REN activities was measured. Data are presented as the mean ± standard deviation from three biological replicates. In (b–d) different letters indicate significant differences among means as determined using one-way ANOVA followed by Tukey’s *post hoc* test (*P* < .05). In (e) significant differences were determined using Student’s *t*-test (^**^P < .01).

To further elucidate the function of the *ZjBRC1*-mediated ABA pathway in regulating lateral shoot branching, we performed expression analysis of *ZjBRC1* and ABA-related genes in different growth stages of jujube lateral main buds. According to the BBCH scale [39], the development of lateral main buds was divided into four stages (Fig. 5a). *ZjBRC1* was expressed at a high level in the dormant lateral main buds (Stage 00) and subsequently decreased from Stage 07 (beginning of bud burst) to Stage 10 (first leaves separating), while *ZjBRC2* showed the opposite profile (Fig. 5b). The expression level of *ZjHB40* was correlated with *ZjBRC1* levels. The expression of *ZjNCED3*, which possessed higher capability to synthesize ABA *in vivo* (Supplementary Data Fig. S2c), decreased sharply from Stage 07 to Stage 10 (Fig. 5b). However, *ZjBRC1* mRNA levels were upregulated in the grown-out lateral main bud with JWB infection (Figs 1e and 5c). We hypothesized that the repression of ZjBRC1 activity by SJP1/2 in turn induced its expression at the mRNA level. Likewise, *ZjBRC1* but not *ZjBRC2* mRNA levels increased in both the *SJP1* and *SJP2* transgenic jujube calli (Fig. 5d). The dual-luciferase reporter assay showed that ZjBRC1 significantly repressed its own expression (Fig. 5e). These results indicated that ZjBRC1 possessed autoinhibitory activity and was negatively correlated with lateral main bud outgrowth.

### ZjBRC1 directly binds to ABA synthesis and receptor genes to control their expression

**Figure 6:**
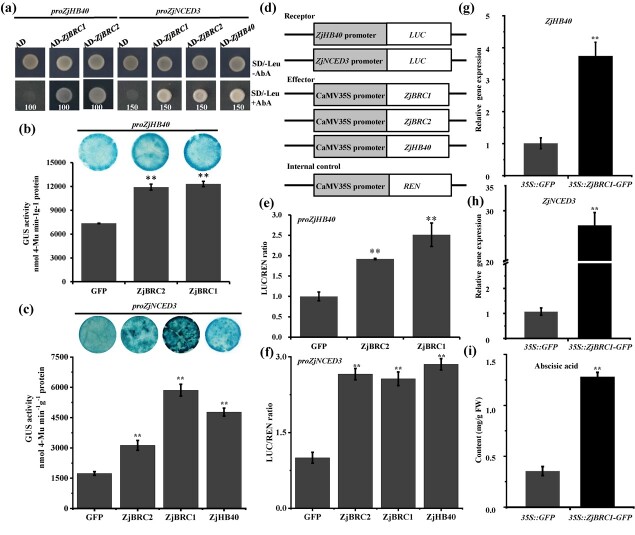
ZjBRC1 bound to *ZjHB40* and *ZjNCED3* promoters to activate their expression and ABA accumulation. (a) Binding of ZjBRC1 and ZjBRC2 to *ZjHB40* and *ZjNCED3* promoters by Y1H assay. (b, c) GUS activity of *ZjHB40* (b) and *ZjNCED3* (c) promoters in transiently co-transformed *N. benthamiana* leaves with *ZjBRC1* and *ZjBRC2*. Representative images of histochemical staining were photographed from at least 10 leaf discs (*R* = 0.5 cm) of three independent leaves. Fluorometric analysis of GUS activity was expressed as nmol 4-methylumbelliferone (Mu) min^−1^ g^−1^ protein. Error bars show standard deviations from three technical replicates (Student’s *t*-test, ^**^P < .01). (d–f) Dual-luciferase assays showing activation of *ZjBRC1* and *ZjBRC2* to *ZjHB40* and *ZjNCED3* promoters in *N. benthamiana* leaves. GFP was used as a negative control. Effector and reporter constructs (d) were co-transformed into *N. benthamiana* leaves, and the ratio of LUC to REN activities (e, f) was measured. Data are presented as the mean ± standard deviation from three biological replicates. (g, h) *ZjHB40* (g) and *ZjNCED3* (h) expression in *ZjBRC1* transgenic calli. *ZjACT1* was used as the internal control. (i) Endogenous ABA contents of *35S::GFP* and *35S::ZjBRC1-GFP* transgenic calli. Error bars show standard deviations from three independent experiments (Student’s *t*-test, ^ **^P < .01).

A prior study suggested that ZjBRC1 repressed bud outgrowth by repressing the auxin efflux channel in jujube [30]. To elucidate whether ZjBRC1 also participated in the regulation of ABA synthesis in lateral main buds, the yeast one-hybrid (Y1H) assay was performed. The results showed that ZjBRC1 and ZjBRC2 directly targeted the *ZjHB40* and *ZjNCED3* promoters in yeast (Fig. 6a). Furthermore, *ZjNCED3* was also a direct ZjHB40 target (Fig. 6a). The *in vivo* interactions between ZjBRC1/2 and the *ZjHB40* or *ZjNCED3* promoter via transient GUS assays in *Nicotiana benthamiana* leaves were then examined. Compared with the GFP control, the GUS activity of the *ZjHB40* and *ZjNCED3* promoters was significantly increased upon cotransformation with 35S::ZjBRC1-GFP or 35S::ZjBRC2-GFP (Fig. 6b and c). ZjHB40 similarly induced the GUS activity of the *ZjNCED3* promoter (Fig. 6c). The dual-luciferase reporter assay confirmed that ZjBRC1/2 and ZjHB40 formed the direct core regulation module to activate *ZjNCED3* expression (Fig. 6d–f). Next, we examined the changes in *ZjNCED3* mRNA and ABA levels in *ZjBRC1* transgenic jujube calli. The qRT–PCR results showed that the expression of *ZjHB40* and *ZjNCED3* was significantly upregulated in *ZjBRC1*-overexpressing transgenic calli compared with *35S::GFP* transgenic calli (Fig. 6g and h). Changes in ABA levels were positively correlated with *ZjHB40* and *ZjNCED3* expression, with a 4-fold increase in *ZjBRC1*-overexpressing transgenic calli (Fig. 6i).

**Figure 7:**
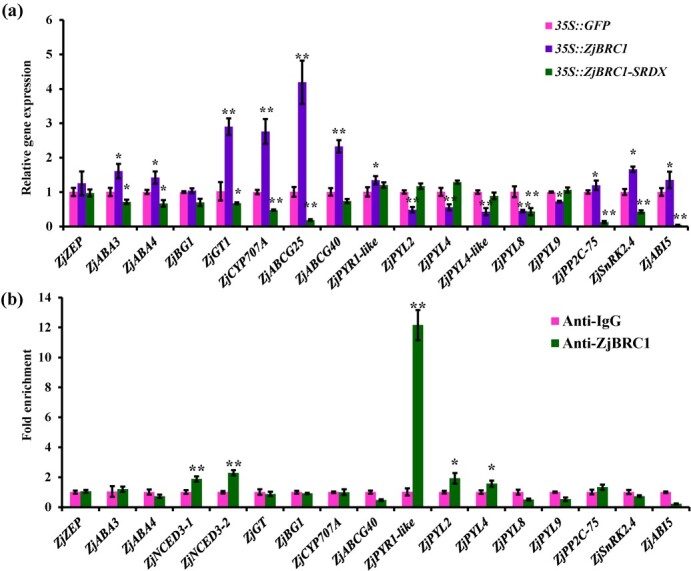
ZjBRC1 widely regulated ABA homeostasis and signalling pathway. (a) Expression of DEGs involved in ABA homeostasis and signalling pathway in *ZjBRC1* transgenic calli. *ZjACT1* was used as the internal control. (b) ChIP–qPCR analysis showed that ZjBRC1 binds to *ZjNCED3* and ABA receptors *ZjPYR1-LIKE/2/4*. Chromatin from *35S::ZjBRC1-GFP* transgenic calli was immunoprecipitated by anti-ZjBRC1. Enrichment of the fragments in the anti-ZjBRC1 group compared with that in the anti-IgG group was determined by qPCR. Means were calculated from three biological samples (^**^P < 0.01 and ^*^P < 0.05, Student’s *t*-test).

Considering that a set of ABA-related genes were differentially expressed (Fig. 2b and c) and the decreased accumulation of ZjBRC1 protein in JWB phytoplasma-infected lateral main buds [30], we investigated the role of *ZjBRC1* in regulating ABA homeostasis and signalling pathways. Compared with the *35S:GFP* transgenic calli, the qRT–PCR results showed that the expression of genes involved in ABA biosynthesis (*ZjABA3* and *ZjABA4*), ABA–glucose conjugation (*ZjGT1*), ABA catabolism (*ZjCYP707A*), ABA transport (*ZjABCG25* and *ZjABCG40*), and the core ABA signalling pathway (*ZjPYR1-LIKE*, *ZjAHG1*, *ZjSNRK2.4* and *ZjABI5*) were significantly upregulated in *ZjBRC1*-overexpressing transgenic calli, while most of them were significantly downregulated in *ZjBRC1-SRDX* transgenic calli (Fig. 7a). To elucidate whether these inducible genes were direct targets of ZjBRC1, we searched for TCP-binding motifs (GGNCCC) across genomic regions. Most of these regions contained at least one putative TCP-binding motif in their promoter or genome sequence (Supplementary Data Fig. S7). The chromatin immunoprecipitation–qPCR assay results showed that ZjBRC1 bound directly to the genomic regions of *ZjNCED3*, *ZjPYR1-LIKE*, *ZjPYL2*, and *ZjPYL4 in vivo* (Fig. 7b). Taken together, these results suggest that ZjBRC1 controls the expression of ABA homeostasis and receptor genes to modulate ABA accumulation and the local ABA response.

### ZjBRC1 forms heterodimers with other CYC/TB1-TCP transcription factors in jujube

**Figure 8:**
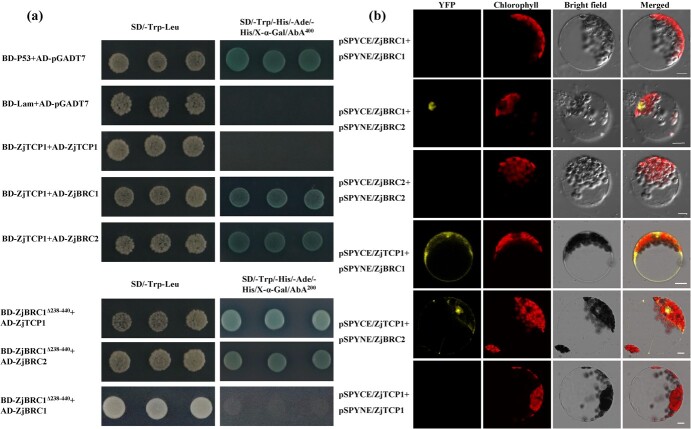
Interaction of ZjBRC1 with jujube CYC/TB1-7 transcription factors. (a) Y2H assay between the CYC/TB1-TCP proteins. The interaction was determined according to the growth of colonies on the SD/−Trp/−His/−Ade/−His/ plates supplemented with 40 μg/ml X-α-Gal and 400 ng/ml AbA. pGADT7-T/BD-p53 and pGADT7-T/BD-Lam were used as the positive and negative controls, respectively. (b) BiFC assays confirmed the interaction of jujube CYC/TB1-TCP transcription factors to form heterodimers in *Arabidopsis* mesophyll protoplasts. Scale bars = 10 μm.

To understand the interactions among the CYC/TB1-TCP transcription factors, dimer formation was determined by the Y2H assay. BD-ZjBRC1 and BD-ZjBRC2 but not BD-ZjTCP1 showed autoactivation activity in yeast on SD/−Trp/−His plates supplemented with 400 ng/ml aureobasidin A (AbA) (Supplementary Data Fig. S8a). Then, specific deletions of ZjBRC1 were constructed (Supplementary Data Fig. S8b). The results showed that BD-ZjBRC1^Δ238–440^ abolished the autoactivation on SD/−Trp/−His plates supplemented with 200 ng/mL AbA (Supplementary Data Fig. S8c); thus, it was used for the Y2H assay. When the prey and bait vectors were cotransformed into yeast, CYC/TB1-TCP transcription factors interacted with each other to form heterodimers, and a homodimer was only observed in ZjBRC1 when the C-terminal 339–440 amino acids were deleted (Fig. 8a and Supplementary Data Fig. S8d). However, the 1–167 amino acid deletion impaired the interaction of ZjBRC1 with ZjBRC2 and ZjTCP1 (Supplementary Data Fig. S8d). These special interaction pairs were also confirmed by bimolecular fluorescence complementation (BiFC) assays (Fig. 8b). YFP fluorescence was observed in the nucleus when ZjBRC1 was coexpressed with ZjBRC2 in *Arabidopsis* mesophyll protoplasts (Fig. 8b). Interestingly, the signal was present in both the nucleus and cytoplasm when ZjBRC1 or ZjBRC2 was coexpressed with ZjTCP1 (Fig. 8b). The formation of heterodimers may be correlated with their biological activity in suppressing lateral bud outgrowth.

## Discussion

Witches’ broom disease caused by members of *Candidatus* (Ca.) Phytoplasma has been identified in many woody plants showing uncontrolled proliferation of axillary buds, such as jujube [8], apple [40], and *Paulownia* [29]. Phytoplasmas are obligate and phloem-restricted bacteria [41], and they interact with both host plants and insect vectors for long periods of time. JWB phytoplasmas have lost many important metabolic pathways that are essential for their survival, and thus they co-opt some genes involved in glycolysis and energy material generation from the host phloem [9]. These obligate parasites attack the dormant jujube lateral main buds to induce the production of more vegetative tissue and prolong their feeding time until winter arrives. Great efforts have been made to obtain knowledge of jujube–phytoplasma interactions [19, 20, 42–44]. However, the complex mechanisms underlying JWB phytoplasma-mediated lateral main bud outgrowth remain largely unknown.

### Jujube witches’ broom phytoplasmas disrupt multiple hormone pathways to stimulate lateral main bud outgrowth in jujube

The phytoplasmic effectors SAP11 and TENGU promote disease susceptibility by disrupting the JA and IAA pathways in the host, which otherwise regulate plant architecture and sterility [11, 45, 46]. We observed rapid expansion and growth within JWB phytoplasma-infected lateral main buds (Fig. 1a), which was consistent with the reprogramming of DEGs related to hormone pathways (Fig. 1d). In our previous research, JWB phytoplasmas disrupted apical dominance by at least partially modulating IAA levels in the infected lateral main buds [30]. Interestingly, DEGs involved in auxin transport, the auxin-activated signalling pathway, and the response to auxin were specifically enriched in concert with the release of the lateral main bud from dormancy (Fig. 1d). Eight auxin efflux genes and five basipetal auxin transport genes were differentially expressed in IGB (Supplementary Data Table S4), and their expression was correlated with the dynamic changes in IAA levels [30]. These results provide a comprehensive understanding of the role of auxin in regulating JWB phytoplasma-mediated bud outgrowth in woody trees.

In addition to IAA, a >2-fold decrease in ABA levels was also observed in the JWB phytoplasma-infected lateral main buds compared with the healthy buds (Fig. 2d). These reductions were consistent with the downregulated DEGs in ABA biosynthesis and transport and the upregulated DEGs in ABA catabolism (Fig. 2b and c). In particular, compared with the healthy dormant buds, the expression of the ABA biosynthesis gene *ZjNCED3* and its potential transcription factor *ZjHB40* (a homologue of *HB21*, *HB40*, and *HB53* from *Arabidopsis*) [35] was significantly downregulated in the JWB phytoplasma-infected lateral main buds. Members of the *ZjCYP70A* family, which lead to increased ABA degradation, were highly expressed in JWB phytoplasma-infected buds. ABA signalling in the infected dormant buds was rapidly altered in response to JWB phytoplasma infection (Fig. 1d). These changes, together with ethylene signalling (Fig. 1d), were negatively correlated with lateral bud dormancy [47–49]. As a negative regulator, ABA controls axillary bud outgrowth in *Arabidopsis* [50, 51], pear [52, 53], grapevine [54], hybrid aspen [55], and maize [56]. Therefore, the regulation of lateral main bud outgrowth by the release of ABA-mediated repression appeared to be conserved in jujube.

The SA and JA pathways play important roles in host plant–phytoplasma interactions [19, 20, 22, 42]. In particular, the JA pathway also participates in lateral branch development; it promotes tiller bud growth in sorghum [57] but dormancy in maize [56]. However, more work is needed to determine whether the JA pathway functions in regulating lateral main bud development and immunity to JWB phytoplasmas and insects in jujube. We did not exclude the possibility that JWB phytoplasmas manipulated sugar signals to control bud outgrowth since a number of DEGs involved in metabolites and energy were enriched during rapid bud expansion (Fig. 1d). Activation of *ZjD27* and *ZjD14* involved in the strigolactone pathway might promote the expression of *ZjBRC1* at transcriptional level in JWB phytoplasma-infected lateral main buds (Fig. 1e). It is worth noting that the cytokinin pathway was not found in buds, which might be caused by the parasitism of JWB phytoplasmas disturbing their transport from the roots to the buds [58].

### Jujube witches’ broom phytoplasmas inhibit ZjBRC1 to modulate ABA levels in the developing lateral main buds

Phytoplasmas alter plant architecture and reproduction through SAP05-SAP11-SAP54-mediated destabilization of multiple developmental regulators (TCP, MADS-box, SPL, and GATA transcription factors) via ubiquitin-dependent and/or ubiquitin-independent pathways [12, 13, 22, 29]. Another virulence factor, TENGU, from the 16SrI group asteris OY phytoplasma, induced witches’ broom and sterility by downregulating auxin-related pathways [45, 46]. Nevertheless, the absence of TENGU-interacting host plant proteins limits the understanding of its pathogenic mechanism. In our previous research, 43 candidate secreted JWB phytoplasma proteins (SJPs) were annotated [59]. *SJP1* and *SJP2* were confirmed to induce an increase in lateral branch number in *N. benthamiana* [30] and *Arabidopsis* (Fig. 3). These phenomena indicated that JWB phytoplasmas recruited *SJP1* and *SJP2* to hijack jujube lateral branch formation. Furthermore, ZjBRC1 was targeted and destabilized by SJP1 and SJP2 in jujube calli [30]. Overexpression of *ZjBRC1* and *ZjBRC2* in *brc1-2* mutants reduced the number of rosette- and cauline-leaf branches; these results were similar to those in the control lines (Fig. 3). Taken together, the results suggest that JWB phytoplasmas employed dual effectors to control ZjBRC1 expression and induce witches’ broom symptoms.

Furthermore, the downregulated ABA pathway in JWB phytoplasma-infected lateral main buds was consistent with the findings in *Arabidopsis brc1-2* mutants [36], indicating that *ZjBRC1* might play a critical role in the JWB phytoplasma-repressed ABA pathway. The *in vivo* interaction revealed that ZjBRC1 formed heterodimers with ZjBRC2 to positively regulate ABA synthesis in jujube calli (Figs 6 and 8). As a downstream target of *ZjBRC1*, *ZjHB40*, which was the only homologue that existed in the jujube genome, directly bound to and activated *ZjNCED3* expression. The *ZjBRC1*–*ZjHB40* module in the regulation of ABA synthesis appeared to be highly conserved in jujube and *Arabidopsis* [35]. In addition, *ZjBRC1* induced ABA catabolism, ABA–glucose conjugation, and ABA transport to promote ABA storage (Fig. 7). Precise regulation was also found in maize buds, in which TB1 targeted *ZEP1* and *VP14* for ABA biosynthesis, *XERICO 1/2* for degradation, *ABCG25* for transport, and genes involved in ABA signalling to maintain bud dormancy [56]. We also observed that three ABA receptors (*ZjPYR1-LIKE*, *ZjPYL2* and *ZjPYL4*) were direct targets of *ZjBRC1* (Fig. 7). These results indicated that the *ZjBRC1* regulatory module was a crucial barrier for JWB phytoplasmas to overcome.

**Figure 9:**
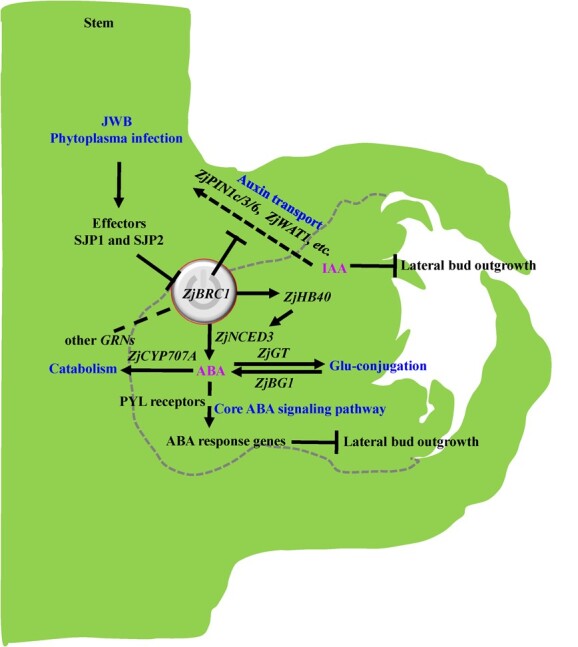
A potential model of JWB phytoplasmas manipulating ZjBRC1 to modulate IAA and ABA levels so as to induce lateral main bud outgrowth. *GRN*s, *BRC1*-dependent gene-regulatory networks [36].

The IAA levels were consistent with ZjBRC1 expression [30] and ABA accumulation in the healthy lateral main bud (Fig. 2d), indicating that high levels of IAA accumulation might activate a positive feedback loop to induce ABA biosynthesis, promoting axillary bud dormancy [37]. This phenomenon was confirmed by the rescue of the bushy appearance of ABA-deficient lines (Fig. 4). However, the long-term effects of ABA on plants might be lethal, since plants expressing *35S:AtBRC1* in *Arabidopsis* [36] and *35S:StBRC1a* potato [60] showed pleiotropic developmental defects and retarded growth. Taken together, the potential pathogenic mechanism of JWB phytoplasmas for inducing the lateral main buds to transition from dormancy to outgrowth was identified (Fig. 9). JWB phytoplasmas secrete the effectors SJP1 and SJP2 into nuclei to target and destabilize ZjBRC1 [30], thus promoting auxin efflux and inhibiting ABA accumulation. These imbalances in hormone levels manipulated the auxin and ABA signalling pathways to release buds from dormancy and activate the expression of genes involved in axillary meristem development. Once the lateral main bud was activated, the axillary meristem developed into one primary bud and two secondary buds, which were displaced upwards by rapid bud expansion. Overall, JWB phytoplasmas hijack *ZjBRC1* to promote lateral main bud outgrowth by modulating IAA and ABA levels in jujube.

### Structural requirements of ZjBRC1 to engineer plants resistant to jujube witches’ broom phytoplasma infection

During JWB phytoplasma infection, *ZjBRC1* serves as a sentinel signal for bud dormancy or outgrowth. How to apply this property to create JWB-resistant varieties is a major challenge for geneticists and breeders. A recent study reported that the SAP05 effector specifically binds to the *Arabidopsis* ubiquitin receptor RPN10 but not the insect vector RPN10 to alter host plant developmental processes [13]. When 38GA39 residues of AtRPN10 were replaced by 38HS39 residues from the insect vector RPN10, *AtRPN10* (38HS39) abolished SAP05 binding and activity [13]. This result provided new insight into how to engineer plants resistant to JWB phytoplasma infection.

Different regions of TCP transcription factors determine the ability to interact with phytoplasma effectors [27], FLOWERING LOCUS T [61, 62], and the bZIP transcription factor FD [63]. ZjBRC1 encodes a 440-amino acid protein with a typical bHLH domain (Supplementary Data Fig. S8b). The N-terminal region of ZjBRC1 is responsible for the interaction with ZjBRC2 and ZjTCP1 (Fig. 8 and Supplementary Data Fig. S8d). The bHLH domain is sufficient for the specificity of SAP11–TCP interactions [27]. However, substitutions of helix, loop, and/or helix sequences within *Arabidopsis* BRC1 abolished the interaction with SAP11_MBSP_ from Maize Bushy Stunt Phytoplasma (MBSP) [27]. In addition, ZjBRC1 also showed autoinhibitory activity (Fig. 5e and Supplementary Data Fig. S8), which might be associated with alternative splicing. In potato, two isoforms of *BRC1a* showed antagonistic functions [60]. The short isoform BRC1a^Short^ interacted with BRC1a^Long^ and reduced its transcriptional activity [60]. Thus, the structural features of *ZjBRC1* should be considered when determining the key domains, residues, or alternative splicing that can block the activities of JWB phytoplasma effectors.

## Materials and methods

### Plant materials

Healthy and JWB-infected 1-year-old primary extension shoots were collected as previously described [30]. The lateral main buds were harvested at the same time and divided into three types based on their development. The JWB-infected 1-year-old primary extension shoots were identified by PCR amplification of the JWB phytoplasma 16S rDNA [64]. At least 100 mg of lateral buds was used for RNA-seq for one biological replicate. The top main buds and secondary shoots of dormant primary extension shoots from 5-year-old ‘Fanchangchangzao’ (*Z. jujuba*) trees were removed by decapitation to promote lateral main bud outgrowth. Stages 00 to 10 of lateral main bud development were observed according to the BBCH scale [39]. Stage 00, Stage 07, and Stage 10 lateral main buds were used to investigate the spatiotemporal expression analysis of CYC/TB1-TCP transcription factors. Transgenic jujube calli carrying 35S::ZjBRC1-GFP and 35S::GFP have been described previously [30]. Seeds of the *brc1-2* mutant (SALK_091920C) were obtained from AraShare (https://www.arashare.cn/index/). All seeds of *Arabidopsis thaliana* and *N. benthamiana* were cold-treated at 4°C for 3 days and then surface-sterilized in 1% sodium hypochlorite solution before sowing on Murashige and Skoog (MS) medium. Seedlings were transplanted into nutrient soil and grown in an artificial climate chamber at 23 ± 1°C under a 16 h/8 h (light:dark) cycle.

### Generation of transgenic *Arabidopsis* lines

*ZjBRC2* coding sequences were fused to the N-terminus of the green fluorescent protein (GFP) gene under the control of the CaMV35S promoter. The recombinant plasmids 35S::SJP1-GFP, 35S::SJP2-GFP, and 35S::ZjBRC1-GFP, which were previously described [30], as well as 35S::ZjBRC2-GFP were transformed into *Rhizobium radiobacter* strain GV3101. Transgenic *Arabidopsis* lines on the Columbia-0 (Col-0) or *brc1-2* background were generated by agroinfiltration using the floral dip method [65]. *35S::GFP* and *35S::GFP/brc1-2* transgenic lines were used as controls. All the lines were confirmed by western blot assay using anti-SJP2 or anti-GFP antibodies.

### ABA treatment and phenotypic analyses of the branches of transgenic lines

For the functional analysis of *ZjBRC1* and *ZjBRC2* in the regulation of lateral branches, three independent *T*_2_ transgenic lines expressing *35S::GFP*, *35S::SJP1*, *35S::SJP2*, *35S::GFP/brc1-2*, *35S::ZjBRC1/brc1-2*, and *35S::ZjBRC2/brc1-2* were grown in MS medium for 12 days and subsequently grown for 4–6 weeks under long-day conditions (16 h light/8 h dark). The shoot branching phenotype and rosette- and cauline-leaf branches (shoots longer than 0.5 cm) were investigated [66]. The main inflorescence became visible 16 days after transplanting from MS medium. All the rosette leaves were removed to display axillary buds. For ABA treatment [35], to avoid the secondary effects, 50 μM ABA solution was added directly to the axils of rosette leaves of 13-day-old transgenic lines once a day after bolting, and branch numbers (shoots longer than 0.5 cm) were counted 15 days after the start of the treatment (*n* = 12). Sterile water treatment was used as a control. Error bars represent the standard deviations (Tukey’s *post hoc* test, *P* < .05).

### Yeast two-hybrid assay and bimolecular fluorescence complementation assays

The coding sequences of *ZjTCP1*, *ZjBRC2*, and *ZjBRC1*, and its deletions *(ZjBRC1*^Δ1–165^, *ZjBRC1*^Δ1–237^, *ZjBRC1*^Δ238–440^, and *ZjBRC1*^Δ339–440^), were cloned into the bait vector pGBKT7. Autoactivation of the recombinant bait vectors in *Saccharomyces cerevisiae* strain Y2HGold was determined on SD/−Trp/−His media supplied with 0, 100, 200, 300, 400, and 500 ng/ml AbA at 30°C for 3–5 days. The interaction analyses were performed by cotransformation of the bait vectors (BD-ZjTCP1, BD-ZjBRC1^Δ1–165^, BD-ZjBRC1^Δ238–440^, BD-ZjBRC1^Δ339–440^, BD-SJP1, and BD-SJP2) with prey vectors as previously described [30] and AD-AtBRC1. The transformed yeast cells were grown on SD/−Ade/−His/−Leu/−Trp media supplied with 40 μg/ml X-α-Gal and 200 or 400 ng/ml AbA at 30°C for 3–5 days. pGADT7-T/BD-p53 and pGADT7-T/BD-Lam were used as the positive and negative controls, respectively.

For bimolecular fluorescence complementation (BiFC) assays, *ZjBRC1*, *ZjBRC2*, and *ZjTCP1* coding sequences were cloned into the pSPYCE vector to generate CE-ZjBRC1, CE-ZjBRC2, and CE-ZjTCP1, respectively. The *in vivo* interactions among the CYC/TB1-TCP transcription factors were investigated by a PEG-Ca^2+^-mediated transformation system of *Arabidopsis* mesophyll protoplasts [67]. All the specific primers for vector construction are listed in Supplementary Data Table S1.

### Ultrastructures of lateral main bud development

The HDB, IDB, and IGB of healthy and JWB-infected primary extension shoots as previously described [30] were fixed in FAA solution [18:1:1 (v/v) 50% ethanol/glacial acetic acid/38% formalin]. Paraffin sections were cut at 9–11 μm thickness and stained with fast green. After fixation in neutral balata, the air-dried slices were placed under a Zeiss light microscope to observe the ultrastructure changes during lateral main bud development and photographed with a ZEN universal imaging system.

### RNA-seq and differential expression analysis

Total RNA was extracted from the HDB, IDB, and IGB of JWB-infected and healthy primary extension shoots using the RNAprep Pure Plant Kit (Tiangen, Beijing, China). Three biological replicates were performed at each bud developmental stage. RNA quantity and quality were assessed using a NanoPhotometer^®^ spectrophotometer (Implen, CA, USA) and a Bioanalyzer 2100 system (Agilent Technologies, CA, USA), respectively. Subsequently, the cDNA library was sequenced on an Illumina NovaSeq platform at Beijing Novogene Bioinformation Technology Co. Ltd., and 150 bp paired-end reads were generated.

The raw reads were filtered and mapped to the jujube genome [68] using HISAT2 (v2.0.5). Novel transcripts were predicted by StringTie (v1.3.3b) [69]. Gene expression levels were quantified by normalized fragments per kilobase per million (FPKM). Differential expression analysis of IDB versus HDB and IGB versus IDB was performed using the DESeq2 R package (1.16.1). Genes with an adjusted *P* value (*padj* value) <0.05 found by DESeq2 and an absolute value of the log2-fold change (FC) ≥ 1 were considered differentially expressed [56].

### Gene coexpression cluster and Gene Ontogeny enrichment analyses

Gene coexpression clustering of a total of 12 376 unique DEGs in the two pairwise comparisons (Supplementary Data Table S2) was performed by H-means clustering, and then DEGs in the four clusters were subjected to Gene Ontology (GO) enrichment analysis by the clusterProfiler R package. GO terms with a *padj* value <0.05 (−lg *padj* > 1.3) were considered significantly enriched. The significantly enriched GO terms and DEGs were visualized by TBtools v1.098661 [70].

### Generation of *35S::ZjBRC1-SRDX* transgenic jujube calli

To prevent the genetic redundancy of the CYC/TB1-TCP transcription factors in the regulation of their downstream gene expression, the coding sequences of *ZjBRC1* were fused to the N-terminus of a codon-optimized repressor domain SRDX (CTGGATCTGGATCTAGAACTCCGTTTGGGTTTCGCTTAA) to produce a gain of repression activity [71]. The desired 35S::ZjBRC1-SRDX vector was transformed into calli by the *Agrobacterium*-mediated sour jujube calli transformation system [30]. The transgenic calli were confirmed by qRT–PCR analysis and then subcultured every 15 days.

### Quantification of ABA

Approximately 100 mg of HDB, IDB, and IGB from JWB-infected and healthy primary extension shoots, *ZjNCED3* and *ZjNCED3L*-overexpressing tobacco leaves, and *35S::ZjBRC1-GFP* transgenic calli were extracted in 1 ml of ice-cold 50% aqueous acetonitrile (vol/vol). All the samples were sonicated for 3 min at 4°C and subsequently extracted for 30 min at 15 rpm and 4°C. After centrifugation, the supernatant was transferred to clean plastic microtubes [72] and used for ABA content measurement by liquid chromatography–electrospray ionization tandem mass spectrometry [64]. Error bars in the figures show the standard deviations from three independent experiments (Student’s *t*-test).

### Yeast one-hybrid, GUS staining and activity, dual luciferase and ChIP–qPCR assays

For the Y1H assay, the ATG upstream regions of *ZjHB40* (LOC107424830, 1636 bp) and *ZjNCED3* (946 bp) were cloned into the HindIII and SalI restriction sites of the pAbAi vector. The coding sequence of *ZjHB40* was cloned into the pGADT7 vector to generate AD-ZjHB40. The interaction of AD-ZjBRC1, AD-ZjBRC2, and AD-ZjHB40 with pAbAi-*proZjHB40* or pAbAi-*proZjNCED3* was carried out as previously described [30]. GUS staining and activity measurements were performed as previously described [73]. For the luciferase (LUC) assay, promoters of *ZjBRC1* (827 bp), *ZjHB40* (1636 bp), and *ZjNCED3* (946 bp) were cloned into the transient expression vector pGreenII 0800-Luc as the reporters. The *ZjBRC1*, *ZjBRC2*, and *ZjHB40* coding sequences were cloned into pGreenII 62-SK as the effectors. Four-week-old *N. benthamiana* leaves were used for coexpression of the reporters and effectors. The empty vector containing GFP was used as a negative control. The firefly luciferase and *Renilla* luciferase activities were quantified with an LB 960 Microplate Luminometer Center (Berthold) using MikroWin software. Transgenic calli expressing *35S::ZjBRC1-GFP* were used for the ChIP–qPCR assay as previously described [30]. Chromatin was immunoprecipitated with anti-ZjBRC1 and anti-IgG antibodies. The relative fold enrichment was generated using the ΔCt (threshold cycle) method. Means were calculated from three biological samples (**P < .01, Student’s *t*-test). All the specific primers for vector construction and the enrichment of TCP-binding motifs in genomic regions are listed in Supplementary Data Table S1.

### Gene expression analysis by quantitative real-time PCR

Total RNA was extracted from lateral main buds and *SJP1/2* and *ZjBRC1* transgenic calli using the RNAprep Pure Plant Kit (Tiangen, Beijing, China). qRT–PCR analyses were performed as previously described [30] using the 2^−ΔCT^ method for tissue expression analysis and the 2^−ΔΔCT^ method for the transgenic calli. *ZjACT1* and *ZjEF1γ* were used as the reference genes for data normalization. qRT–PCR primers used to determine the expression patterns of the *ZjBRC1*, *ZjBRC2*, *ZjHB40*, *ZjNCED3*, and ABA-related genes are listed in Supplementary Data Table S1. Error bars show the standard deviations from three independent experiments (Student’s *t* test).

## Acknowledgements

This work was supported by the National Natural Science Foundation of China (31971687 and 32002007), the Anhui Province Key Research and Development Program (202004a06020008), the Natural Science Foundation of Anhui Province (2008085QC127), and the Natural Science Foundation of Anhui Provincial Department of Education (KJ2019A0186).

## Author contributions

J.S., Q.S., and F.M. planned and designed the research; S.Z., F.M., M.C., N.Z., M.D., and J.Z. performed the experiments; Y.Y., Y.L., C.M., X.Z., C.G., X.H., Z.Z., W.C., and T.L. conducted the transformation; J.S., Q.S., and F.M. analysed the data; F.M. wrote the manuscript; and J.S. and Q.S. revised the manuscript.

## Data availability

All relevant data generated or analyzed are included in the manuscript and the supporting materials.

## Conflict of interest

The authors declare that there are no conflicts of interest.

## Supplementary data

Supplementary data is available at *Horticulture Research* online.

## Supplementary Material

Web_Material_uhad148
