# Supplementary material for: Jujube witches’ broom phytoplasmas inhibit ZjBRC1-mediated abscisic acid metabolism to induce shoot proliferation
Source: Hortic Res. 2023 Jul 24;10(9):uhad148. doi: 10.1093/hr/uhad148 (PMC10483173; doi:10.1093/hr/uhad148)
Supplement: Web_Material_uhad148 [file web_material_uhad148.zip › SUPPLEMENTAL INFORMATION.docx]

**SUPPLEMENTAL INFORMATION**


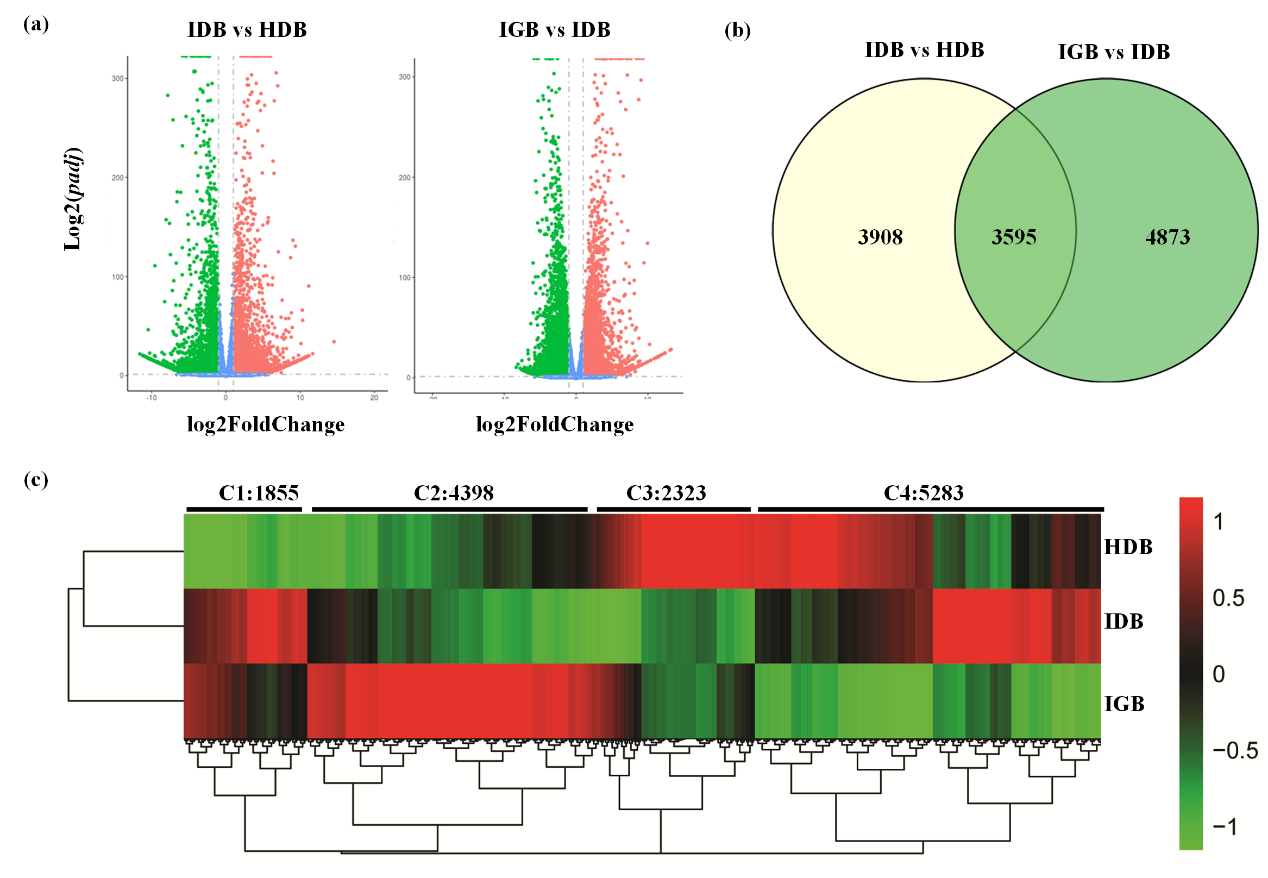
 **Figure S1.** RNA-seq analysis of lateral main bud development in JWB phytoplasma-infected jujube plants. (a) The total differentially expressed genes (DEGs) between IDB vs HDB and IGB vs IDB. Genes with an adjusted *p*-value (*padj*) ≤0.05 and an absolute value of log2-fold change (FC) ≥ 1 were considered differentially expressed. (b) Venn diagrams showing the total differentially expressed genes (DEGs) between IDB vs HDB and IGB vs IDB. (c) Co-expression clusters of DEGs during lateral main bud development.


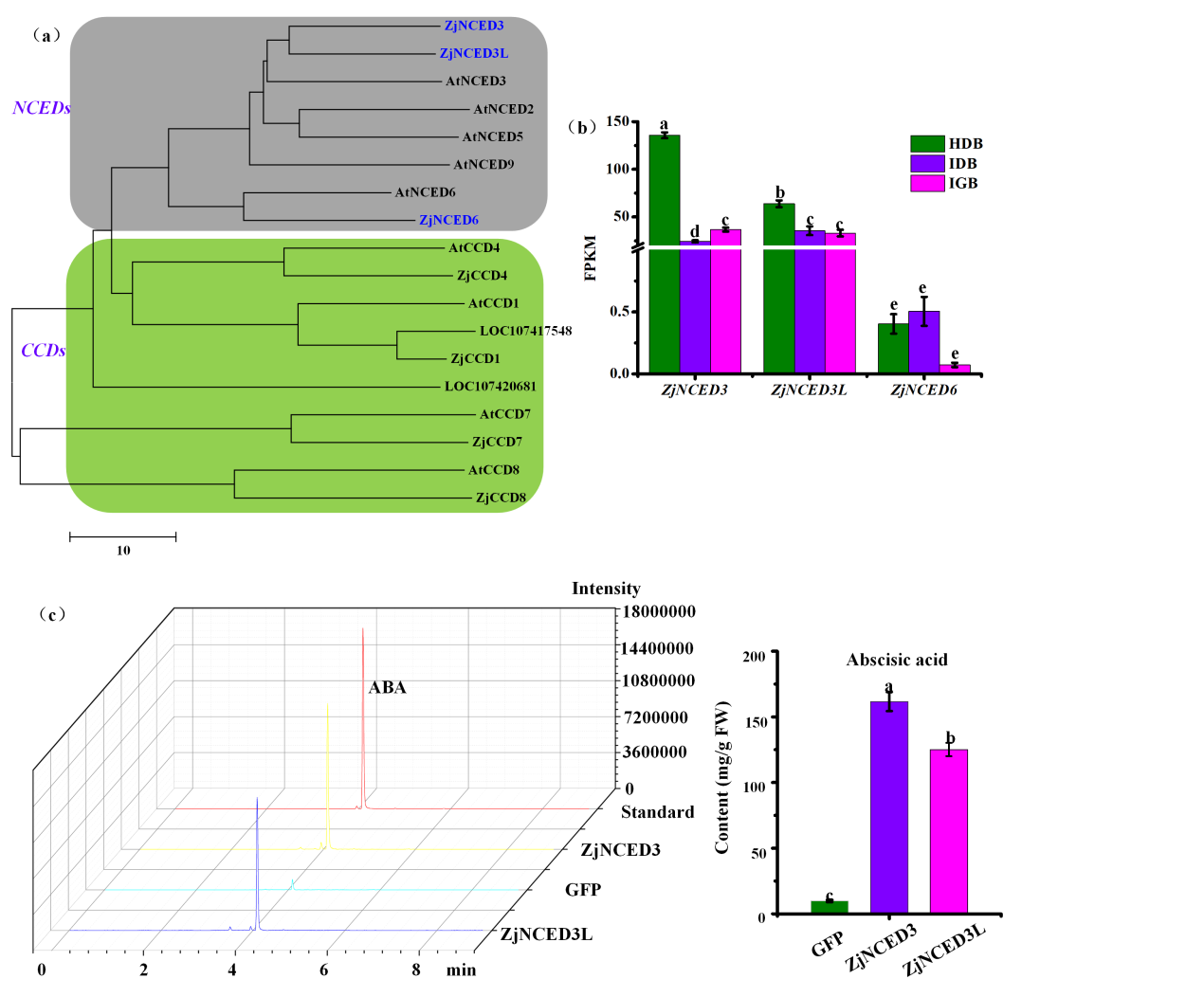


**Figure S2.** Phylogenetic analysis, expression and enzymatic activity of ZjNCED3. (a) Phylogenetic analysis of NCED proteins from jujube and Arabidopsis using the maximum likelihood method. The phylogenetic tree was constructed using MEGA7 software with 1000 bootstraps replicates. (b) Total expression levels of *NCED* genes using the FPKM value in the healthy and JWB phytoplasma-infected buds. (c) *In vivo* enzymatic activities of ZjNCED3 and ZjNCED3L. *ZjNCED3* and *ZjNCED3L* were transiently co-expressed in *N. benthamiana* leaves. Error bars show the SDs from three independent experiments (Tukey’s post-hoc test, *P*< 0.05).


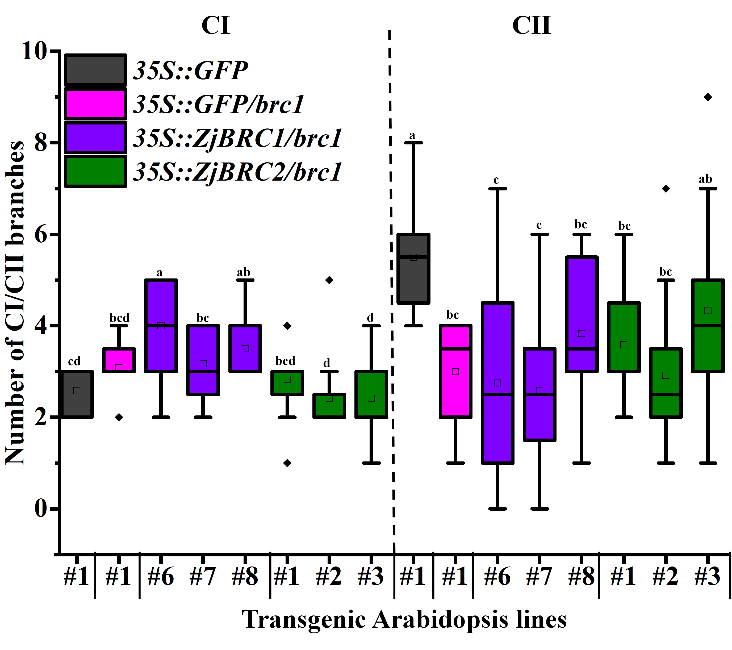


**Figure S3.** Cauline-leaf branches of *35S::ZjBRC1/brc1-2* and *35S::ZjBRC2/brc1-2* transgenic lines at 30 days after transplanting from MS medium. Error bars represent the standard deviations (n=12, Tukey’s post-hoc test, *P*<0.05).


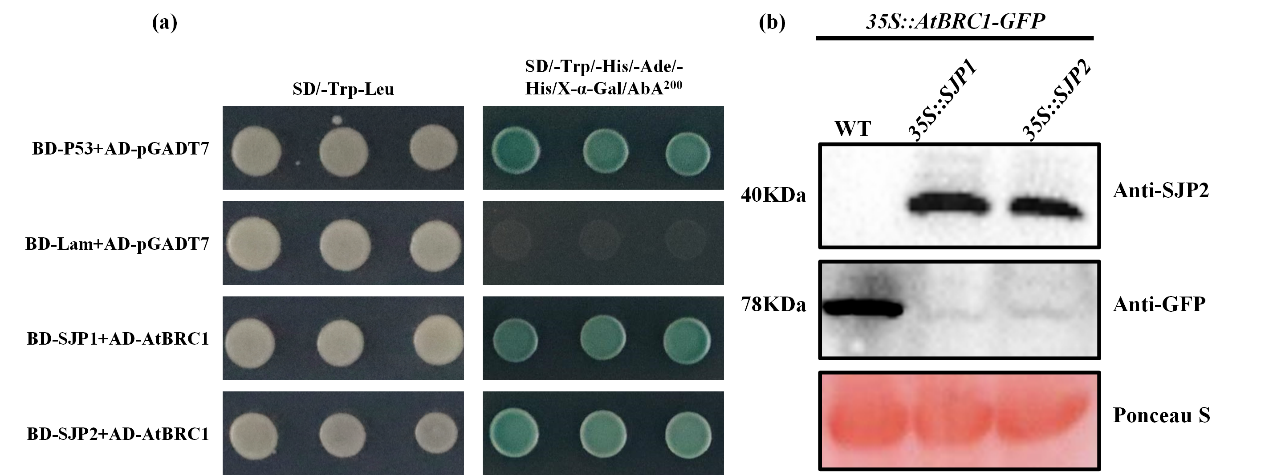


**Figure S4.** JWB phytoplasma effectors SJP1 and SJP2 interacted with and destabilized Arabidopsis AtBRC1. (a) Interaction of SJP1 and SJP2 effectors with AtBRC1. AbA, aureobasidin A. (b) The destabilization of AtBRC1 by SJP1 and SJP2 effectors.


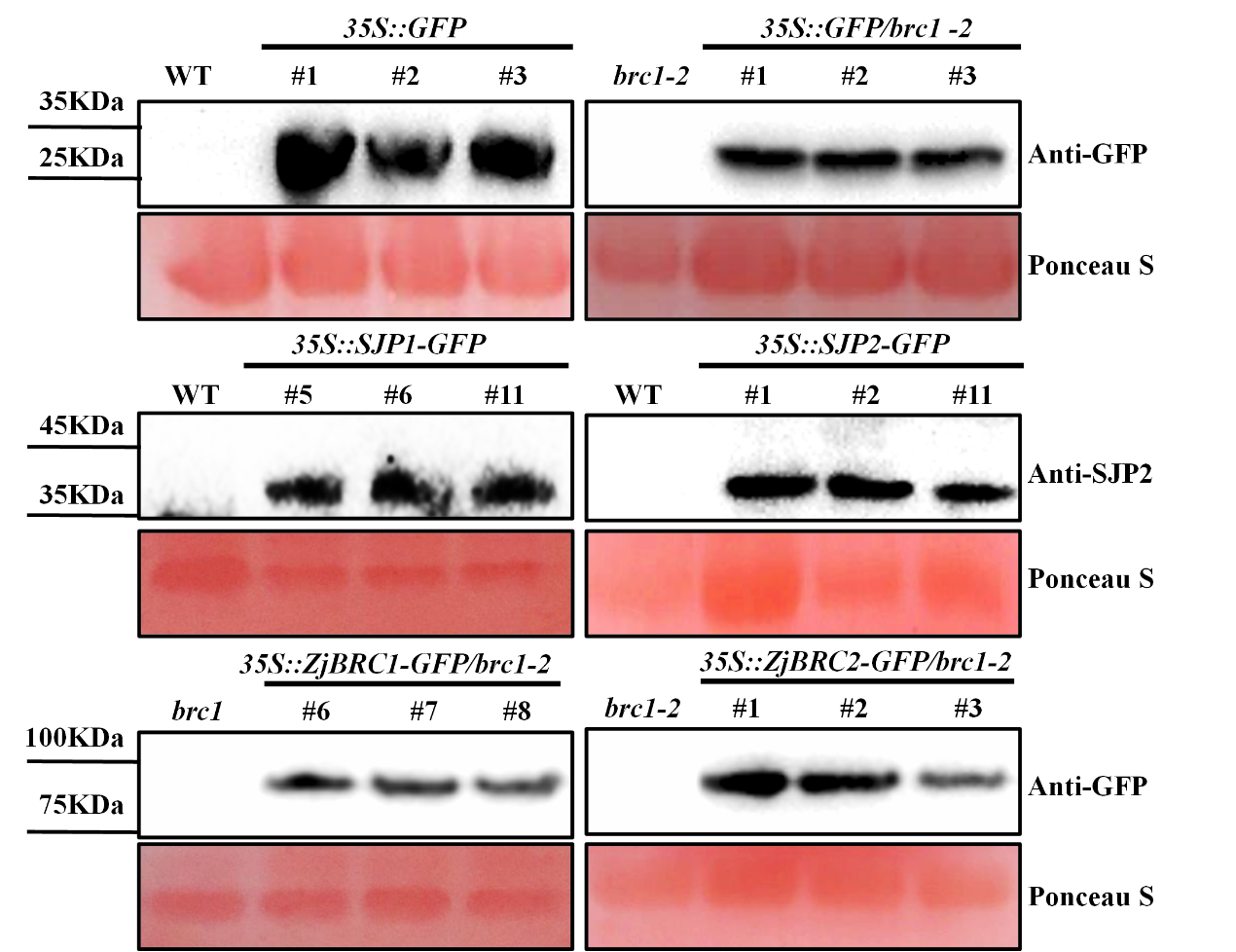


**Figure S5.** The identification and expression of the *35S::SJP1-GFP*, *35S::SJP2-GFP* and *brc1-2* mutants carrying *35S::GFP*, *35S::ZjBRC1-GFP* and *35S::ZjBRC2-GFP* transgenic Arabidopsis lines. *35S::GFP* and *35S::GFP*/*brc1-2* transgenic Arabidopsis were used as the control.


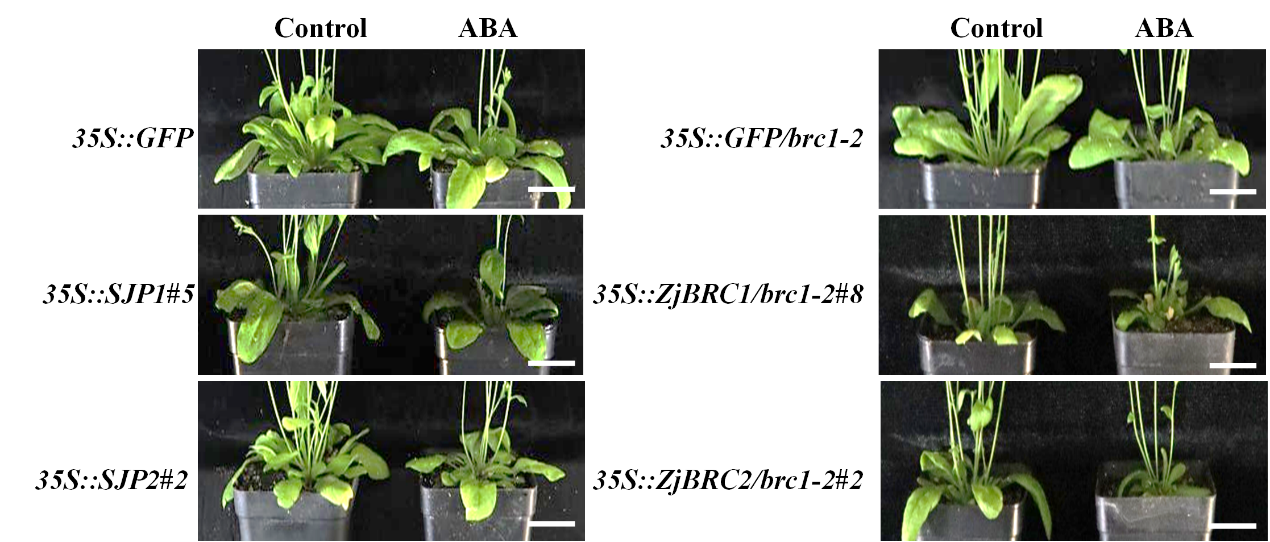


**Figure S6** Close-up views of rosette leaves and branches in the transgenic lines carrying *35S::SJP1*, *35S::SJP2*, *35S::ZjBRC1/brc1-2* and *35S::ZjBRC2/brc1-2* treated with or without 50 μM ABA.


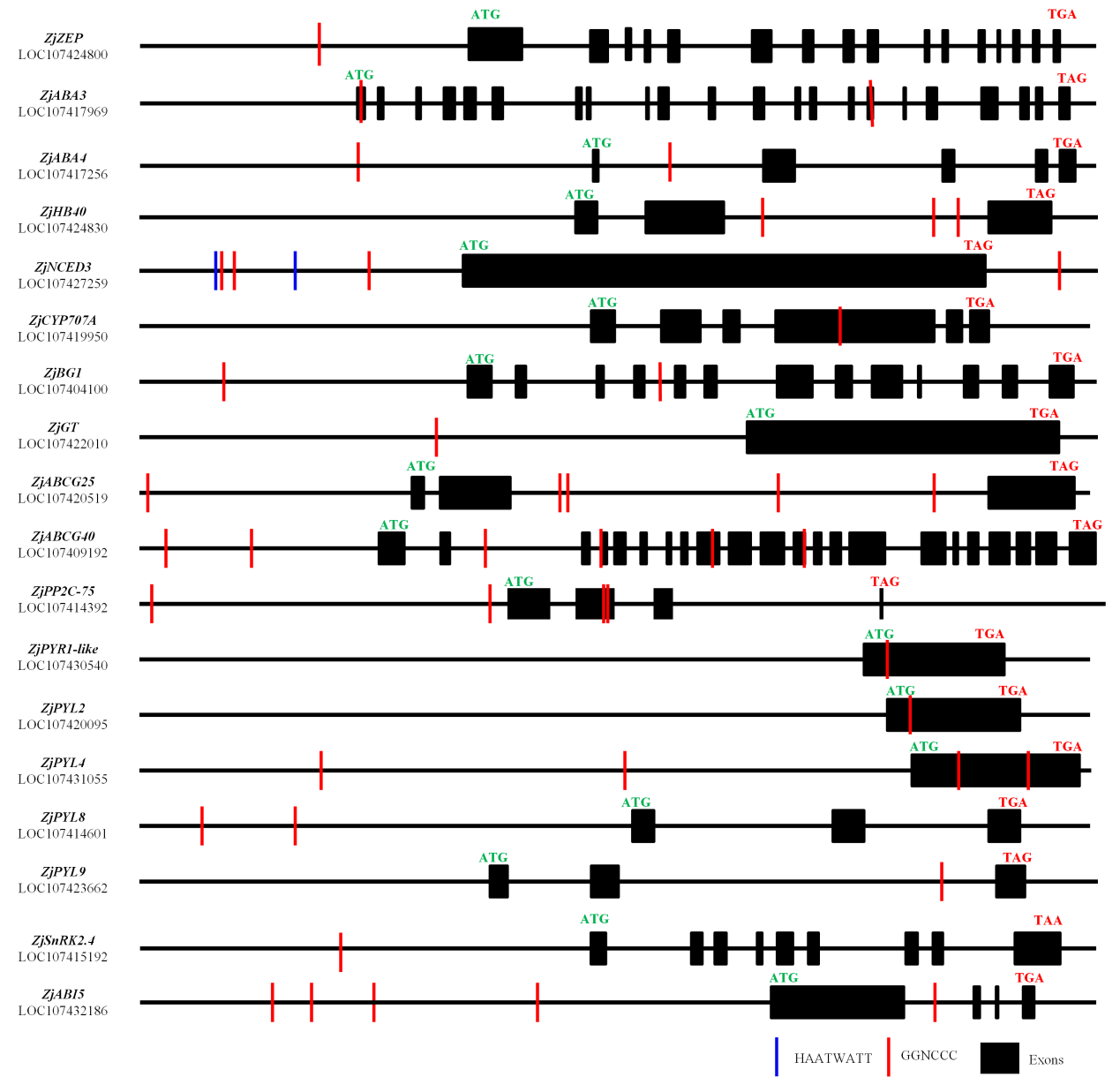


**Figure S7.** TCP-binding motifs in a 2-kb region upstream of the ATG start codon and genomic regions of ABA-related DEGs. The red lines indicate the TCP-binding motif (GGNCCC) and the blue lines indicate the HB40-binding motif (HAATWATT).


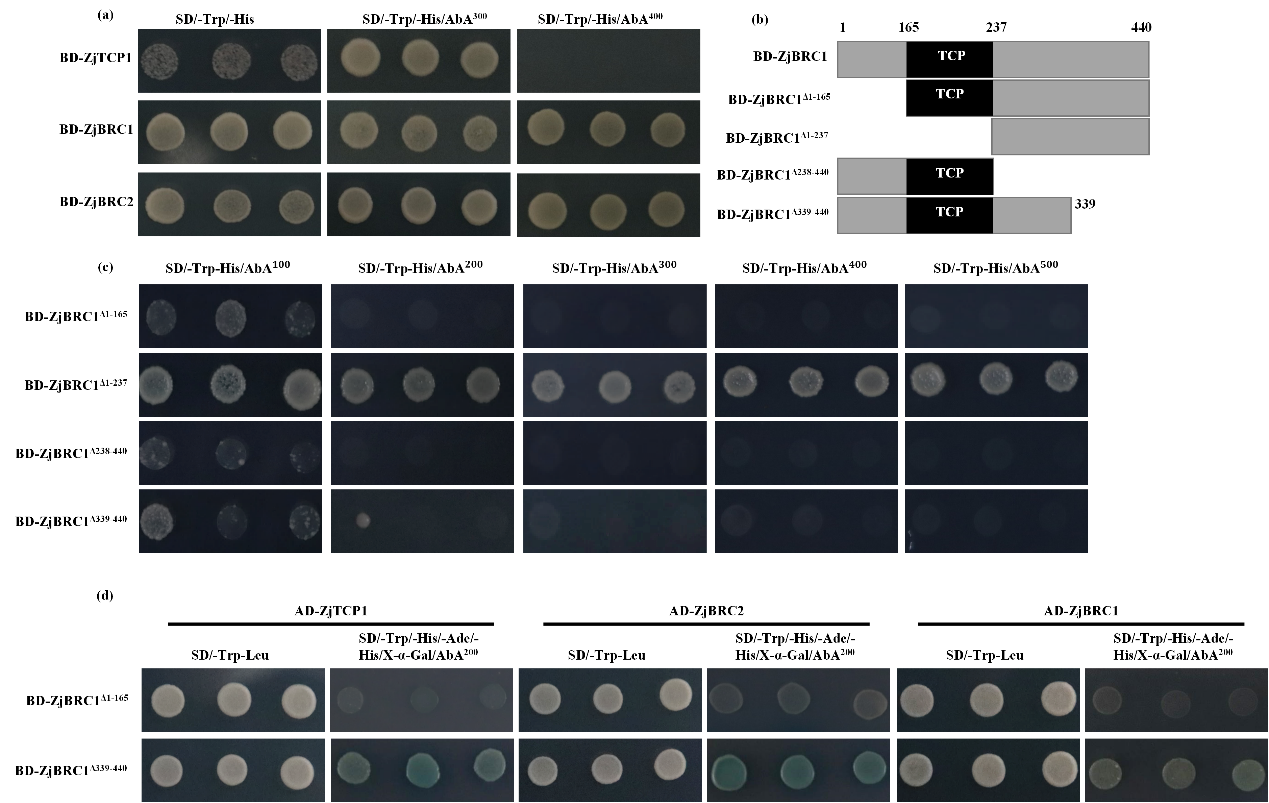


**Figure S8.** Autoactivation test and interaction of ZjBRC1 with jujube CYC/TB1-TCP transcription factors. (a) Autoactivation test of the CYC/TB1-TCP proteins. Bait autoactivation was determined according to the growth of colonies on the SD/-Trp /-His plates supplemented with different content of AbA. AbA, aureobasidin A. (b, c) The deletions (b) and autoactivation test (c) of ZjBRC1. (d) The interaction of BD-ZjBRC1^Δ1-165^ and BD-ZjBRC1^Δ339-440^ with jujube CYC/TB1-TCP proteins by yeast two-hybrid assay.

Table S1 List of primers used in this study.

Table S2 DEGs between IDB vs HDB (7503) and IGB vs IDB (8468).

Table S3 GO-enrichment analysis of 12,376 unique DEGs.

Table S4 DEGs involved in ABA and IAA pathway.
