# Supplementary figures and images for: Jujube witches’ broom phytoplasmas inhibit ZjBRC1-mediated abscisic acid metabolism to induce shoot proliferation
Source: Hortic Res. 2023 Jul 24;10(9):uhad148. doi: 10.1093/hr/uhad148 (PMC10483173; doi:10.1093/hr/uhad148)

(a)

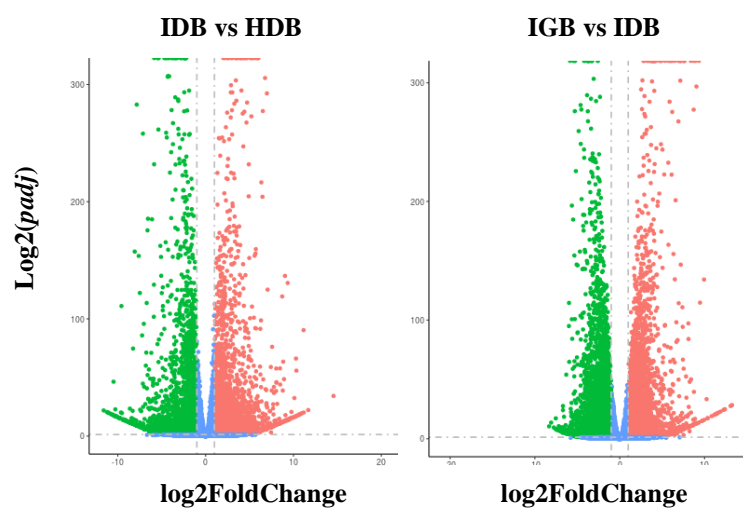

(b)

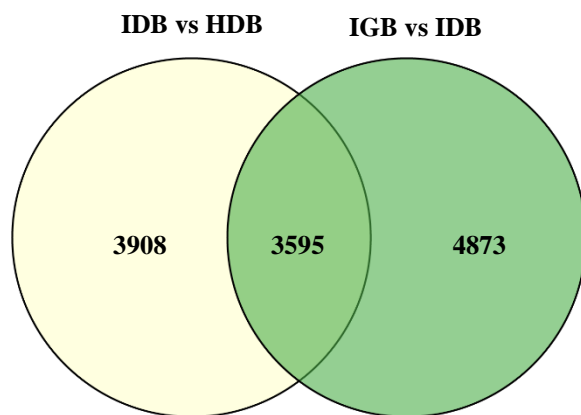

(c)

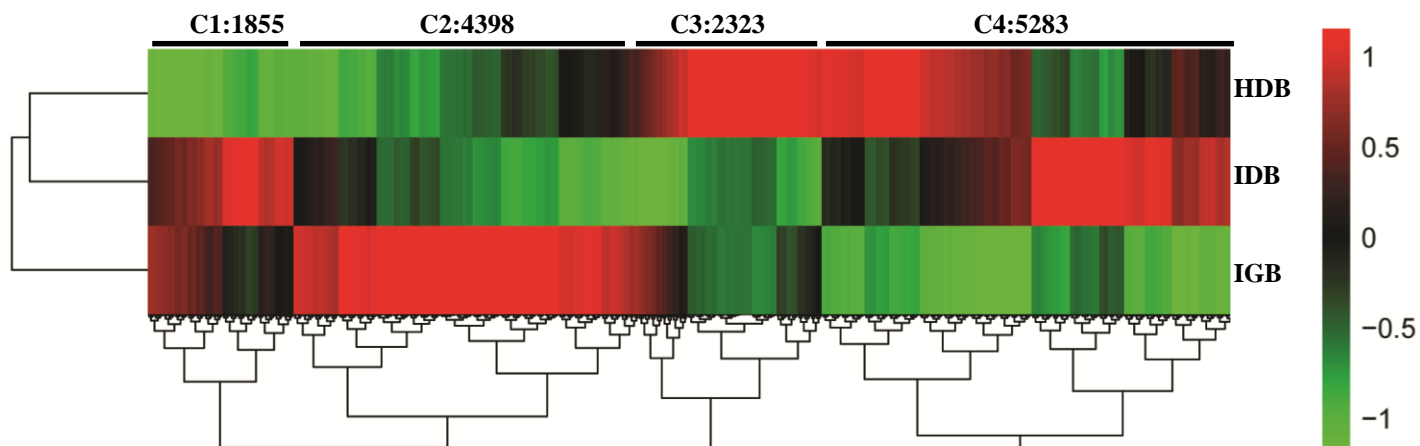

Supplement: Web_Material_uhad148 [file web_material_uhad148.zip › figure S1.pdf]

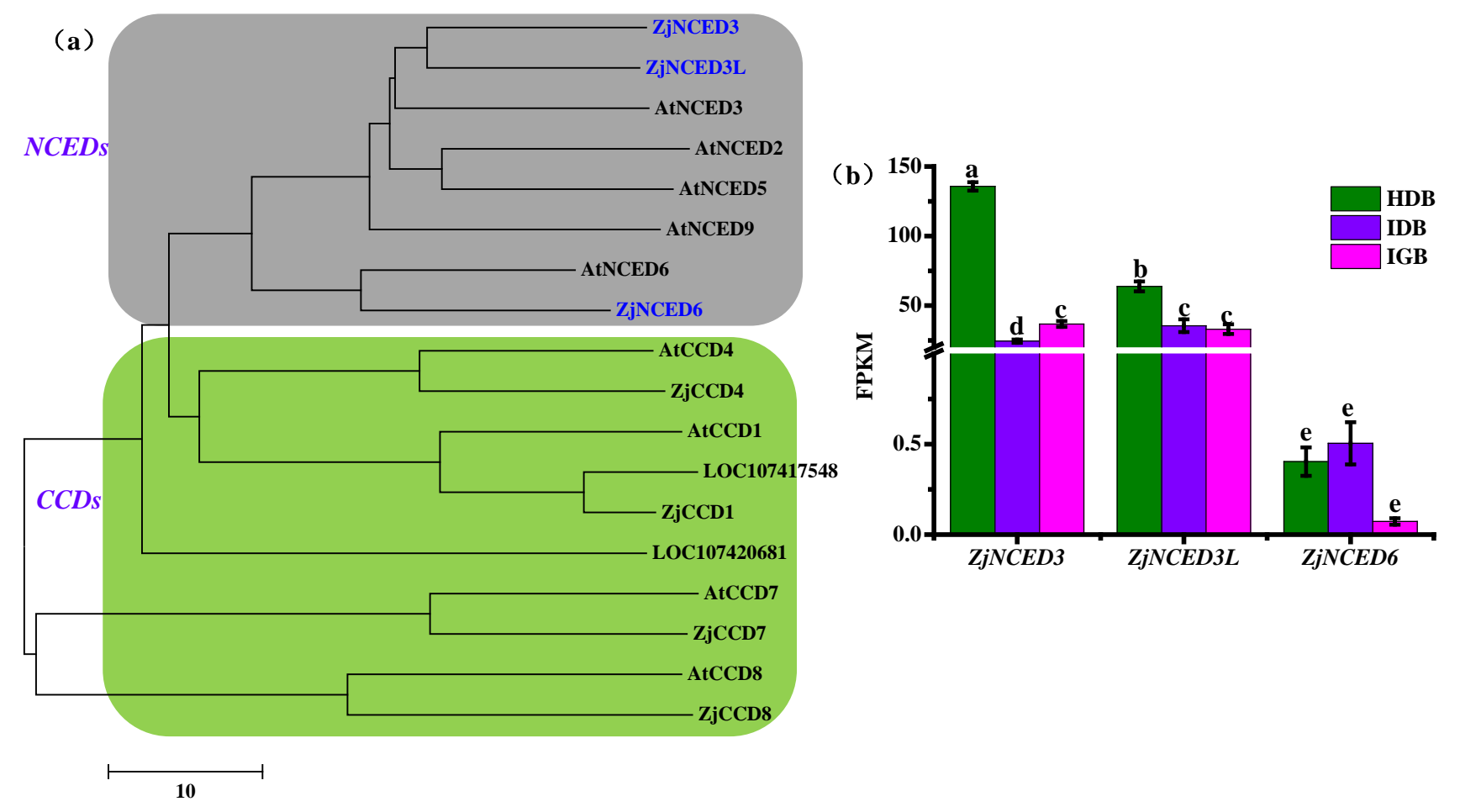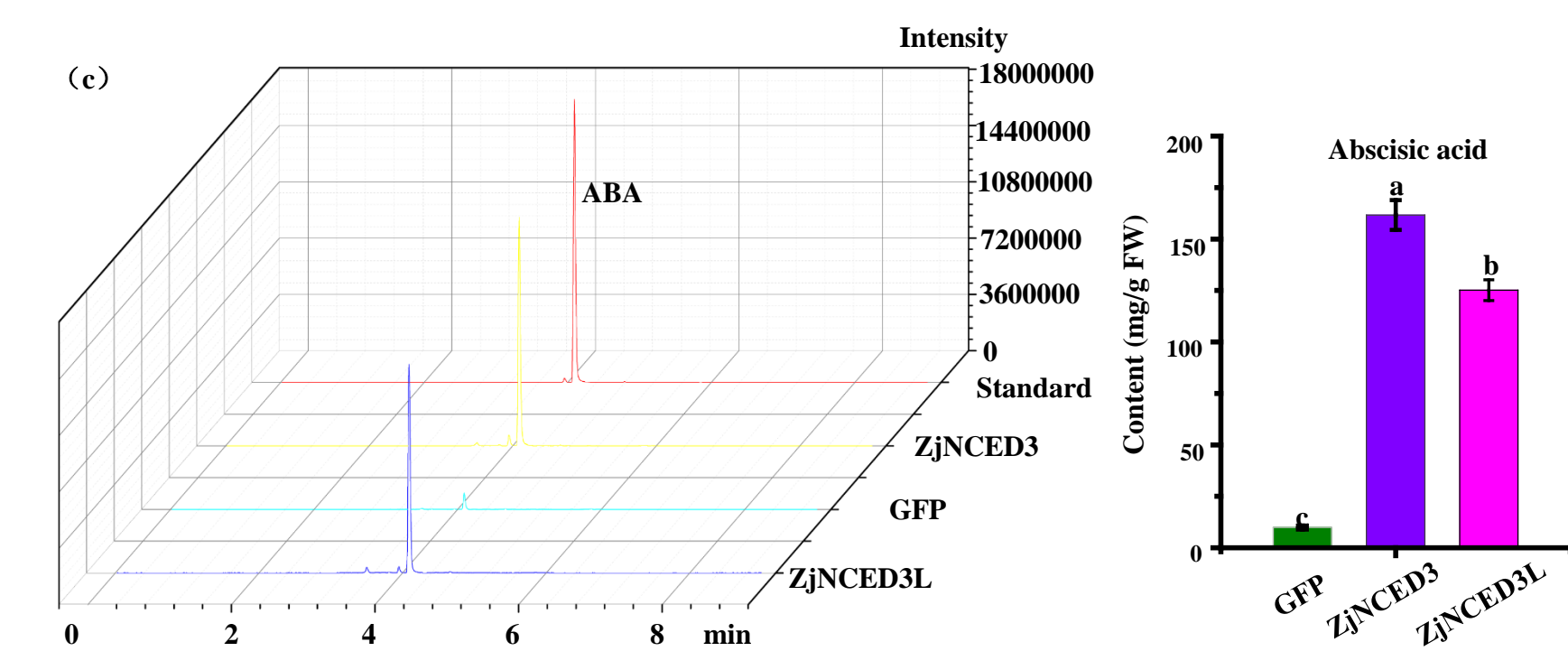

Supplement: Web_Material_uhad148 [file web_material_uhad148.zip › figure S2.pdf]

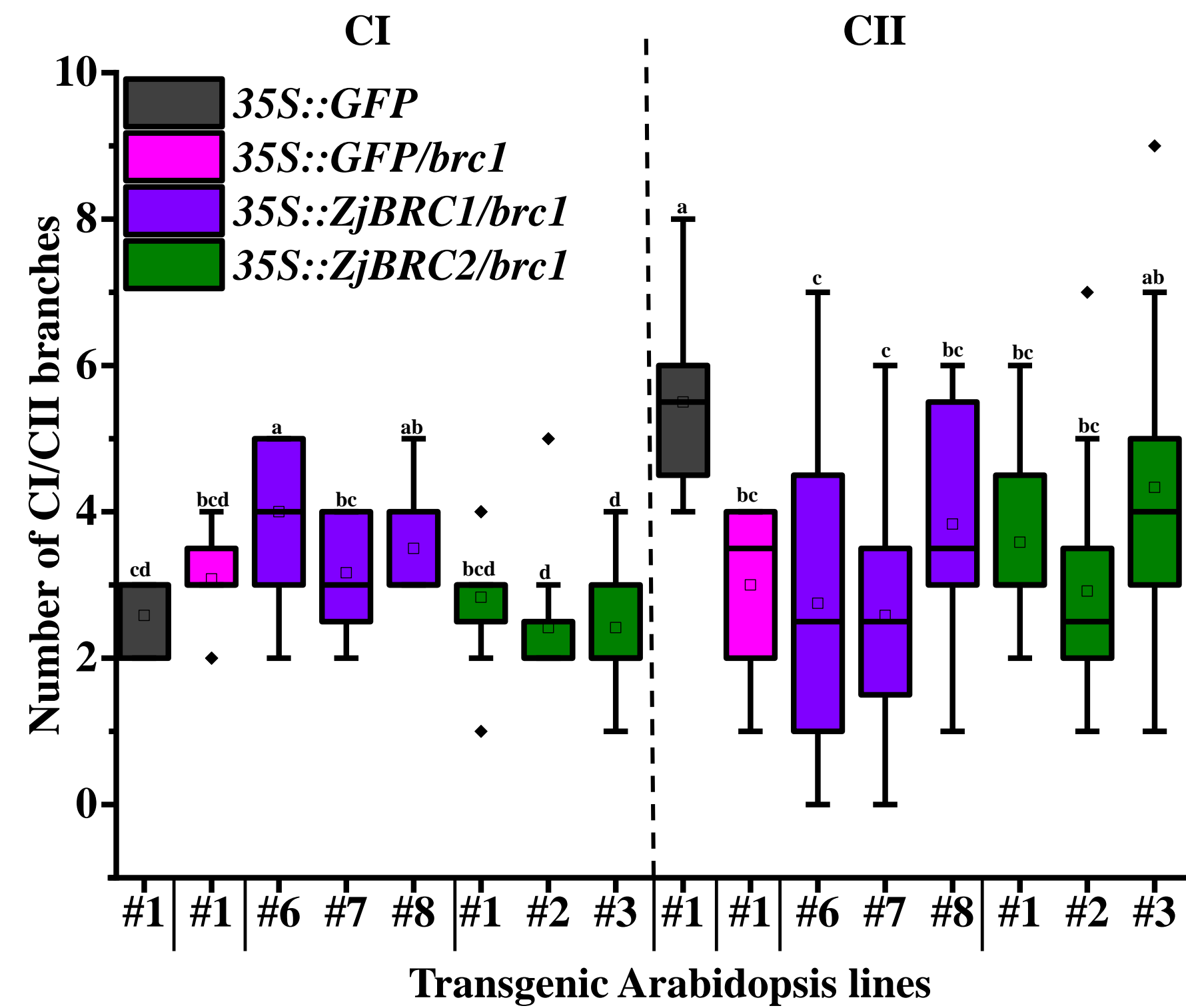

Supplement: Web_Material_uhad148 [file web_material_uhad148.zip › figure S3.pdf]

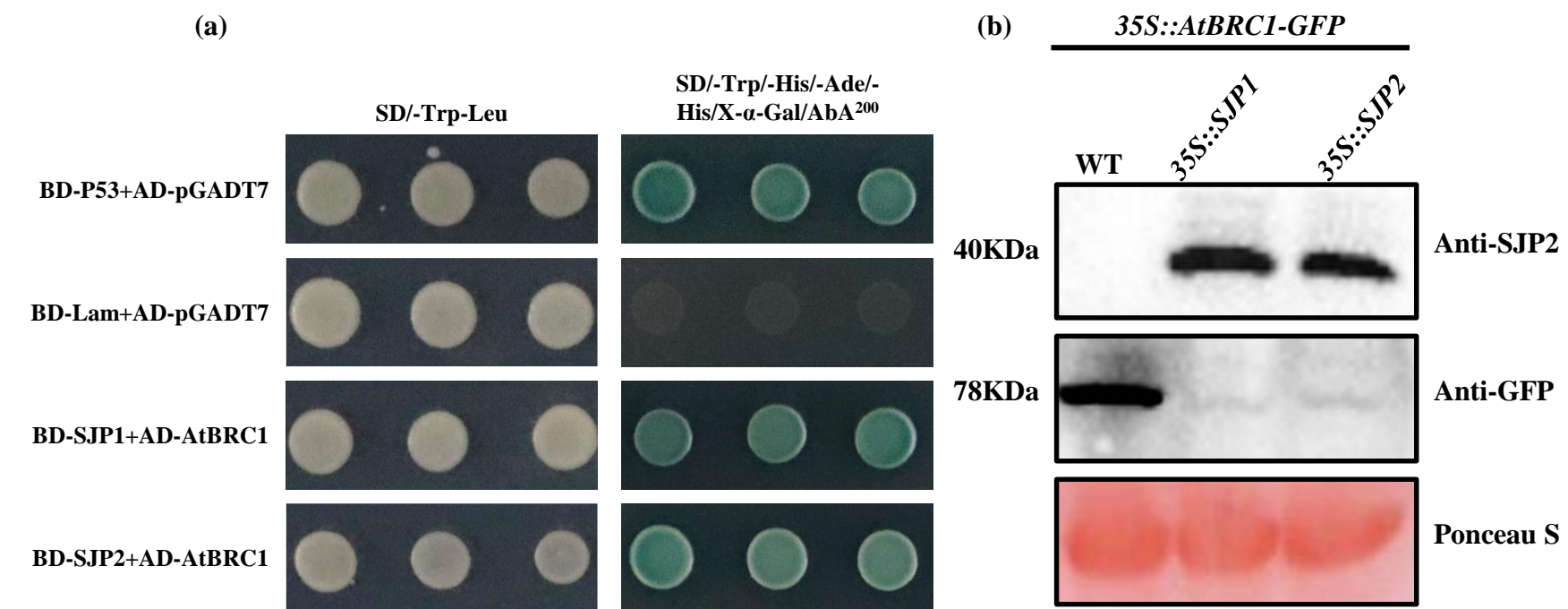

Supplement: Web_Material_uhad148 [file web_material_uhad148.zip › figure S4.pdf]

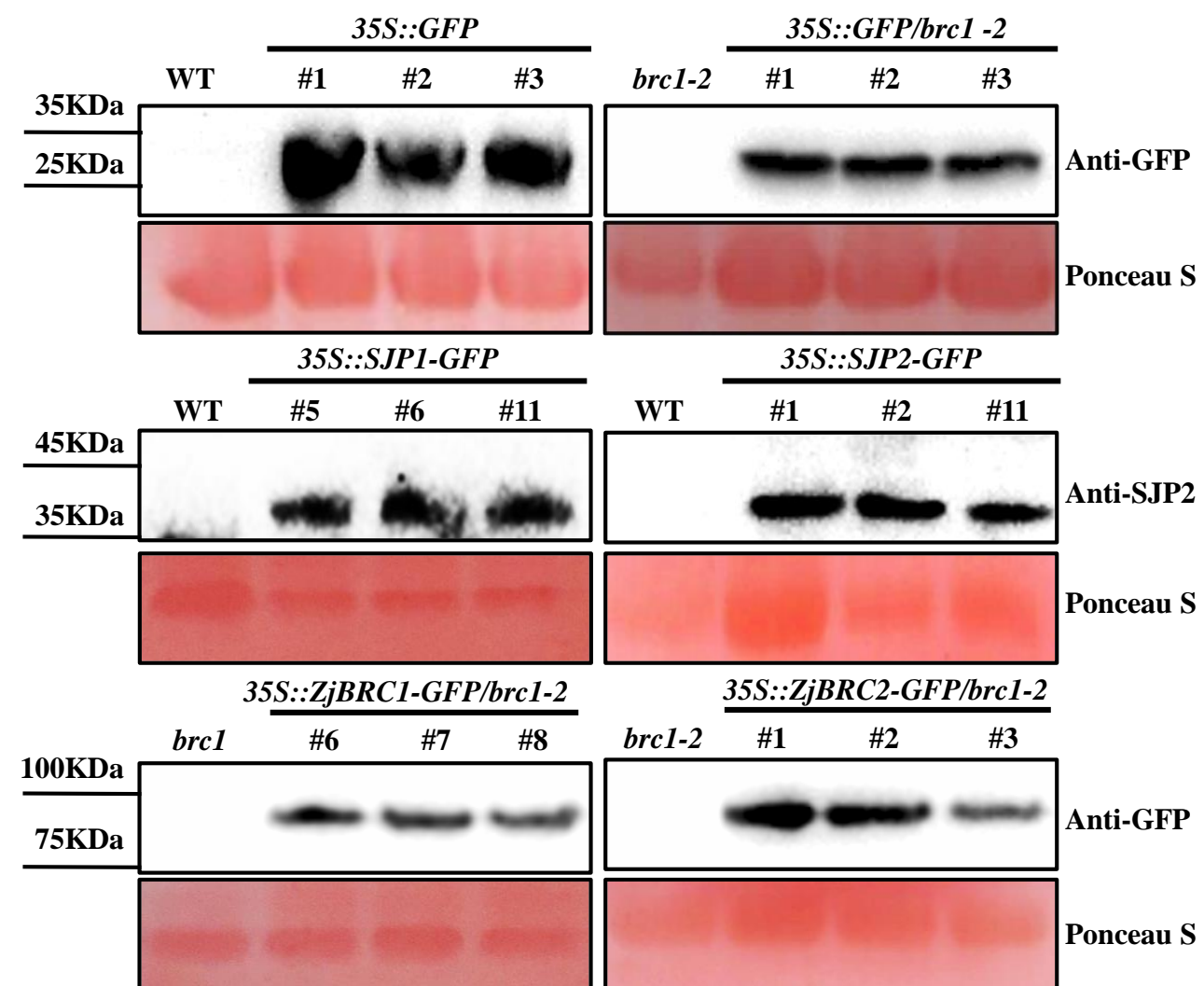

Supplement: Web_Material_uhad148 [file web_material_uhad148.zip › figure S5.pdf]

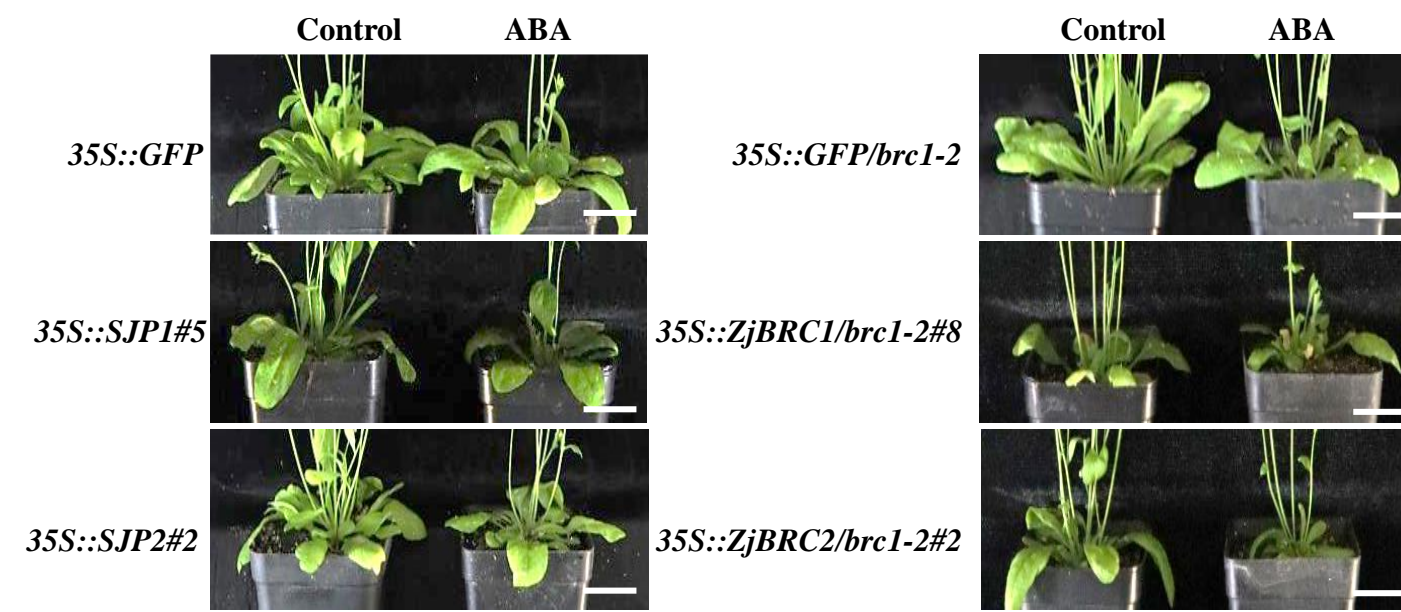

Supplement: Web_Material_uhad148 [file web_material_uhad148.zip › figure S6.pdf]

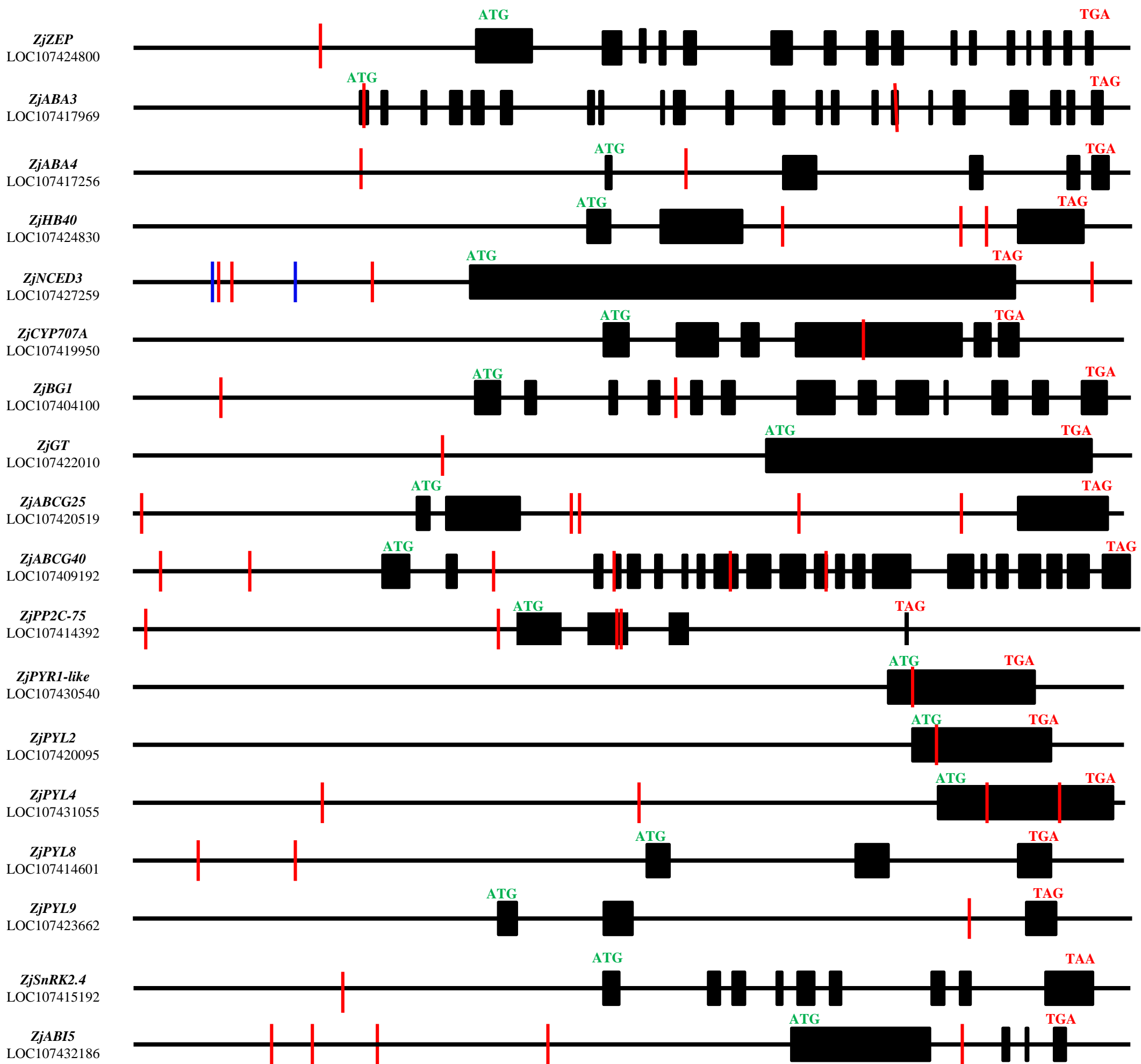

HAATWATT GGNCCC Exons

Supplement: Web_Material_uhad148 [file web_material_uhad148.zip › figure S7.pdf]

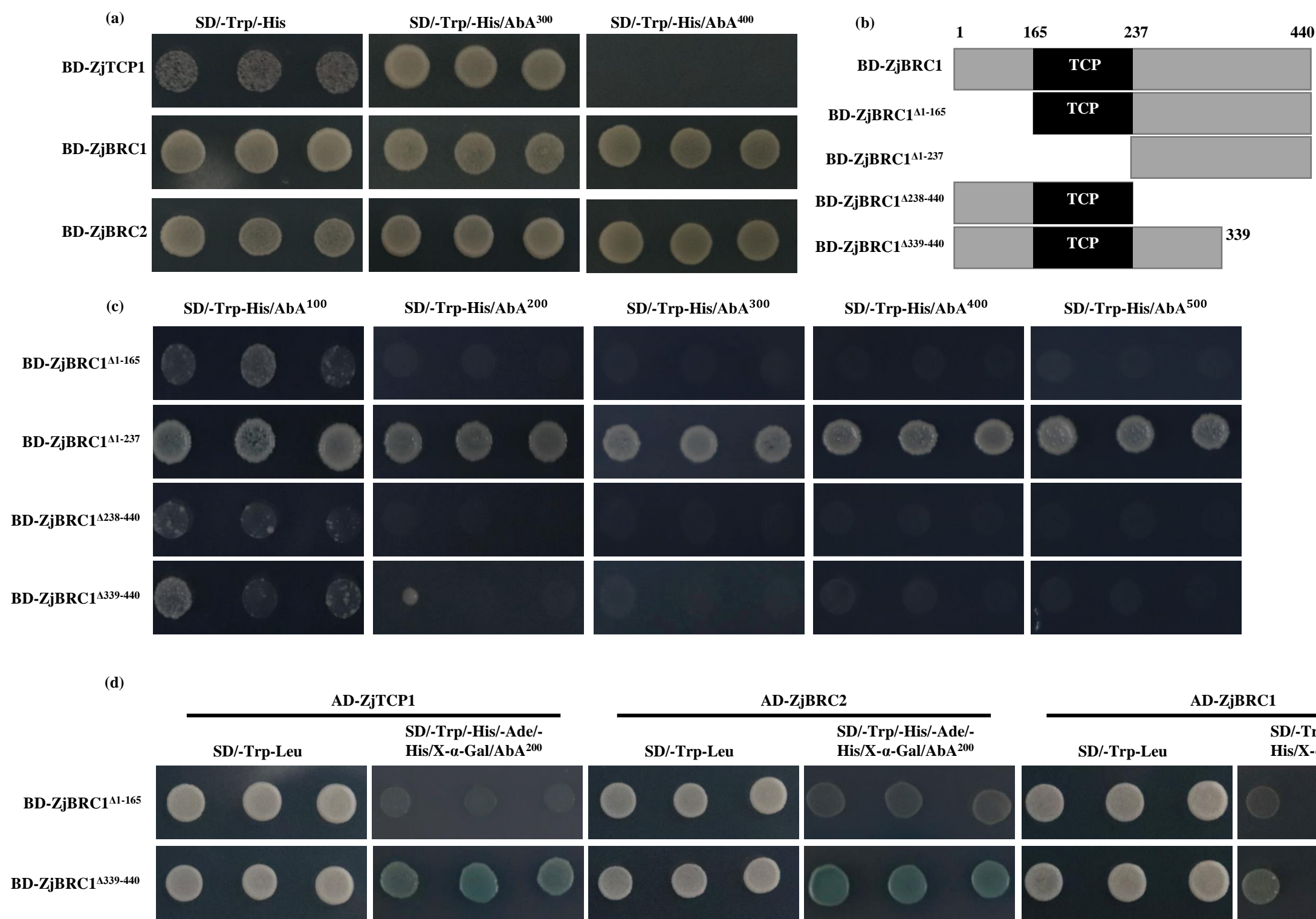

Supplement: Web_Material_uhad148 [file web_material_uhad148.zip › figure S8.pdf]
